# Comprehensive analysis of the transcriptional expressions and prognostic value of S100A family in pancreatic ductal adenocarcinoma

**DOI:** 10.1186/s12885-021-08769-6

**Published:** 2021-09-16

**Authors:** Hong-Bin Li, Jian-Li Wang, Xiao-Dong Jin, Lei Zhao, Hui-Li Ye, Yan-bei Kuang, Yong Ma, Xiang-Yan Jiang, Ze-Yuan Yu

**Affiliations:** 1 School of Life Science and Engineering, Lanzhou University of Technology, Lanzhou, 730050 China; 2 Gansu Provincial Cancer Hospital, Gansu Provincial Academic Institute for Medical Research, Lanzhou, 730050 China; 3 Institute of Modern Physics, Chinese Academy of Sciences, Lanzhou, 730030 China; 4 Cui-ying Biomedical Research Center, Lanzhou University Second Hospital, Lanzhou, 730030 China; 5 Department of General Surgery, Gansu Provincial Cancer Hospital Lanzhou University Second Hospital, No.82 Cui-ying Men, Lanzhou, 730030 Gansu Province China

**Keywords:** S100A, Pancreatic ductal adenocarcinoma, Biomarkers, Prognosis, Bioinformatics

## Abstract

**Background:**

Pancreatic ductal adenocarcinoma (PDAC) remains a treatment-refractory malignancy with poor prognosis. It is urgent to identify novel and valid biomarkers to predict the progress and prognosis of PDAC. The S100A family have been identified as being involved in cell proliferation, migration and differentiation progression of various cancer types. However, the expression patterns and prognostic values of S100As in PDAC remain to be analyzed.

**Methods:**

We investigated the transcriptional expressions, methylation level and prognostic value of S100As in PDAC patients from the Oncomine, GEPIA2, Linkedomics and cBioPortal databases. Real-time PCR was used to detect the expressions of S100A2/4/6/10/14/16 in four pancreatic cancer cell lines and pancreatic cancer tissues from PDAC patients undergoing surgery. To verify the results further, immunohistochemistry was used to measure the expression of S100A2/4/6/10/14/16 in 43 PDAC patients’ tissue samples. The drug relations of S100As were analyzed by using the Drugbank database.

**Results:**

The results suggested that, the expression levels of S100A2/4/6/10/14/16 were elevated to PDAC tissues than in normal pancreatic tissues, and the promoter methylation levels of S100A S100A2/4/6/10/14/16 in PDAC (*n* = 10) were lower compared with normal tissue (*n* = 184) (*P* < 0.05). In addition, their expressions were negatively correlated with PDAC patient survival.

**Conclusions:**

Taken together, these results suggest that S100A2/4/6/10/14/16 might be served as prognostic biomarkers for survivals of PDAC patients.

**Supplementary Information:**

The online version contains supplementary material available at 10.1186/s12885-021-08769-6.

## Introduction

Pancreatic ductal adenocarcinoma (PDAC) remains one of the most malignant tumors with poor prognosis and high mortality. In recent years, its morbidity and mortality have increased significantly, and the 5-year survival rate is < 1% [1]. Due to the lack of early screening indicators, early PDAC is difficult to detect and therefore, spreads rapidly to surrounding organs, making it one of the deadliest cancers [2]. At present, the treatment of PDAC is very limited, and almost only surgical resection can provide a realistic opportunity of survival [3]. Thus, there is an urgent need to identify novel and valid markers to predict the progression and prognosis, and to understand the mechanism of PDAC. Calcium ions are the important factor that controls the balance between signal transduction, cell survival, and metabolism, whose dysregulation is thought to be closely related to cancer development [4]. The S100A family is a class of small-molecule calcium-binding proteins, which are identified only in vertebrates [5]. Many of their members are abnormally expressed in various cancer types, which are widely involved in cell proliferation, migration and differentiation during cancer development [6]. For instance, five calcium-binding proteins (S100A2/7/8/9/10) have been identified as overexpressed in gastric cancer, which might be important for gastric tumorigenesis [7]. Gupta S et al. reported that, the abnormal over-expression of S100A4 observed in cancer tissues are associated with an increase in tumor grade in prostate adenocarcinoma [8]. Analogously, Wang et al. found that, the expression level of S100A11 in colorectal cancer tissue was increased following stage progression of tumor progress [9].

Aberrant expressions and the prognostic values of some members of S100As family have been found in previous studies. However, to date, roles of S100As in the occurrence and development of PDAC remains largely elusive. Here, for the first time, we have used bioinformatics analysis to address this problem by analyzing the expression profiles, methylation level and mutations of different S100As family members and some relations with clinical parameters in PDAC patients. We investigated the expression of S100As in four PDAC cell lines. In addition, PDAC tissues and their matched adjacent normal pancreatic tissues from three PDAC patients undergoing surgery were collected, the expression levels of some S100As were measured using real-time PCR, and further verification was performed by immunohistochemistry in 43 cases of pancreatic cancer samples. Furthermore, we also analyzed genes, which have the similar expression pattern as S100As in PDAC tissues, and predicted the functions and pathways of related genes. This method based on the analysis of different omics levels, and the verification of clinical samples make full use of bioinformatics data, which provides a complementary means for finding potential biomarkers of PDAC.

## Materials and methods

### Oncomine database analysis

Oncomine is a web-based cancer microarray database and integrated data-mining platform. The gene expression data referenced during the study are available in Oncomine website (www.oncomine.com). In our study, transcriptional expressions of 16 different S100As members between different types of cancer and the corresponding normal controls were acquired from Oncomine database. The differences in transcriptional expression were compared through students’ t test. The relevant parameters are as follows: data type: mRNA, *p* value: 1E-4, fold change: 2, gene rank: 10%.

### GEPIA 2 analysis

GEPIA2 (Gene Expression Profiling Interactive Analysis) was used to analyze transcriptional expressions of 16 different S100As members between different types of cancer and the corresponding adjacent normal control. The relevant parameters are as follows: |Log2FC| Cutoff:1, q-value Cut off:0.01, match the TCGA normal and Genotype Tissue Expression (GTEx) data, statistical methods: ANOVA. GEPIA 2 were also used to analyze Overall Survival of PDAC patients. Cutoff-High (%):50, Cutoff-Low (%):50.

### UALCAN database analysis

UALCAN is an online tool for in-depth analyses of TCGA gene expression data [10]. The transcript levels of S100As in PDAC samples were analyzed using the UALCAN program. We also performed survival analysis (OS) based on the expression status of S100As signature and plot a Kaplan-Meier curve by using UALCAN.

### LinkedOmics analysis

LinkedOmics website (http://www.linkedomics.org), which includes multi-omics data from all 32 TCGA cancer types, was used to screen the positively correlated significant genes of S100As, and the parameters are described as follows. Cancer cohort: TCGA_PAAD; search dataset: RNAseq; target dataset: RNAseq; statistical method: Pearson correlation test. Based on the score of person correlation, select the top 25 genes for further research.

### cBioPortal

We retrieved the genomic profiles of S100As members, which contained mutations and clinical data from cBioportal (http://www.cbioportal.org/). The relevant parameters are as follows: Selected Studies: Pancreatic Adenocarcinoma (TCGA, PanCancer Atlas)(184 total samples); Select Genomic Profiles: mutation, mRNA expression z-score relative to diploid (RNA Seq V2 RSEM), the z-score threshold ±1.8. Select Patient/Case Set: samples with mRNA data (RNA Seq V2)(177). Gene list: S100A2, S100A4, S100A6, S100A10, S100A14, S100A16.

### Methylation analysis

MEXPRESS (https://mexpress.be/index.html) is a data visualization tool designed to easily visualize TCGA expression, DNA methylation and clinical data, and the relationship between them [11]. We analyzed the methylation levels of S100A2, S100A4, S100A6, S100A10, S100A14, S100A16 using the MEXPRESS database.

### Drugbank analysis

The DrugBank database (https://www.drugbank.ca/) is a unique bioinformatics and cheminformatics resource that combines detailed drug data with comprehensive drug target information [12]. The DrugBank (version 5.1.6) database was used to analyze drug information targeting S100As.

### Protein–protein interaction network construction

Linkedomics database was used to search for significant genes positively correlated to S100As in pancreatic cancer samples. The significant genes were used for further PPI construction based on STRING online database (http://string-db.org). Furthermore, GO analysis and KEGG pathway enrichment of the screened S100As were carried out based on the Metascape [13].

### Cell culture

Human pancreatic cell line hTERT-HPNE, human pancreatic cancer lines PANC-1, CFAPC-1, MIA PaCa-2 and ASPC-1 were obtained from the cell bank of Chinese Academy of Sciences (Shanghai, China). All of the cells were maintained in high glucose DMEM media supplemented with 10% fetal bovine serum, and were incubated at 37 °C in a humidified atmosphere containing 5% CO_2_.

### RNA preparation and real-time PCR

Total RNA was isolated by Trizol reagent (Invitrogen, USA), and quantified by 260/280 ratios determined using Nanodrop. cDNA was synthesized by PrimeScript RT Master Mix (Takara, China) from 500 ng of RNA. Real-time PCR was performed using TB Green Premix (Takara, China). Real-time PCR amplifications were performed on the QuantStudio 5 System. The expression levels of S100As were normalized to GAPDH in each individual sample. The relevant primers were listed in supplementary Table S1.

### Immunohistochemistry

Immunohistochemistry (IHC) was performed using standard procedures described as previous study [14]. The intensity was scored as “0,” “1,” “2,” and “3”; that was negative, weak, moderate, and strong staining, respectively. The positively stained cells percentage was scored as “0 to 100” The final IHC score was obtained by product between intensity score and stained area. The primary antibodies were used for this study: goat anti-rat S100A2/4/6/10 dilution 1:200 (Abcam); goat anti-rat S100A14 and S100A16 dilution 1:200 (Proteintech). And a score of less than 150 is considered low expression, and a score of 150 or more is considered high expression.

### Statistical analysis

SPSS version 26.0 (IBM, Armonk, NY, USA) was used to perform statistical analyses. Comparison between groups was made using the Student’s t-test, and the mean ± standard derivation for normally distributed continuous data, as the median (interquartile range, Q25 - Q75) for abnormally distributed continuous data. Moreover, categorical data was analyzed using the Pearson chi-square test or Fisher’s exact test and presented as percentages or proportions. Kaplan-Meier analyses was used to analyze overall survival rate between different groups with a log-rank test was used to calculate the overall cumulative probability, and the difference was presented as hazard ratio (HR) value and *P* value. *P*-values < 0.05 were considered significant.

## Results

### Expression patterns of S100As in pancreatic cancer

**Fig. 1:**
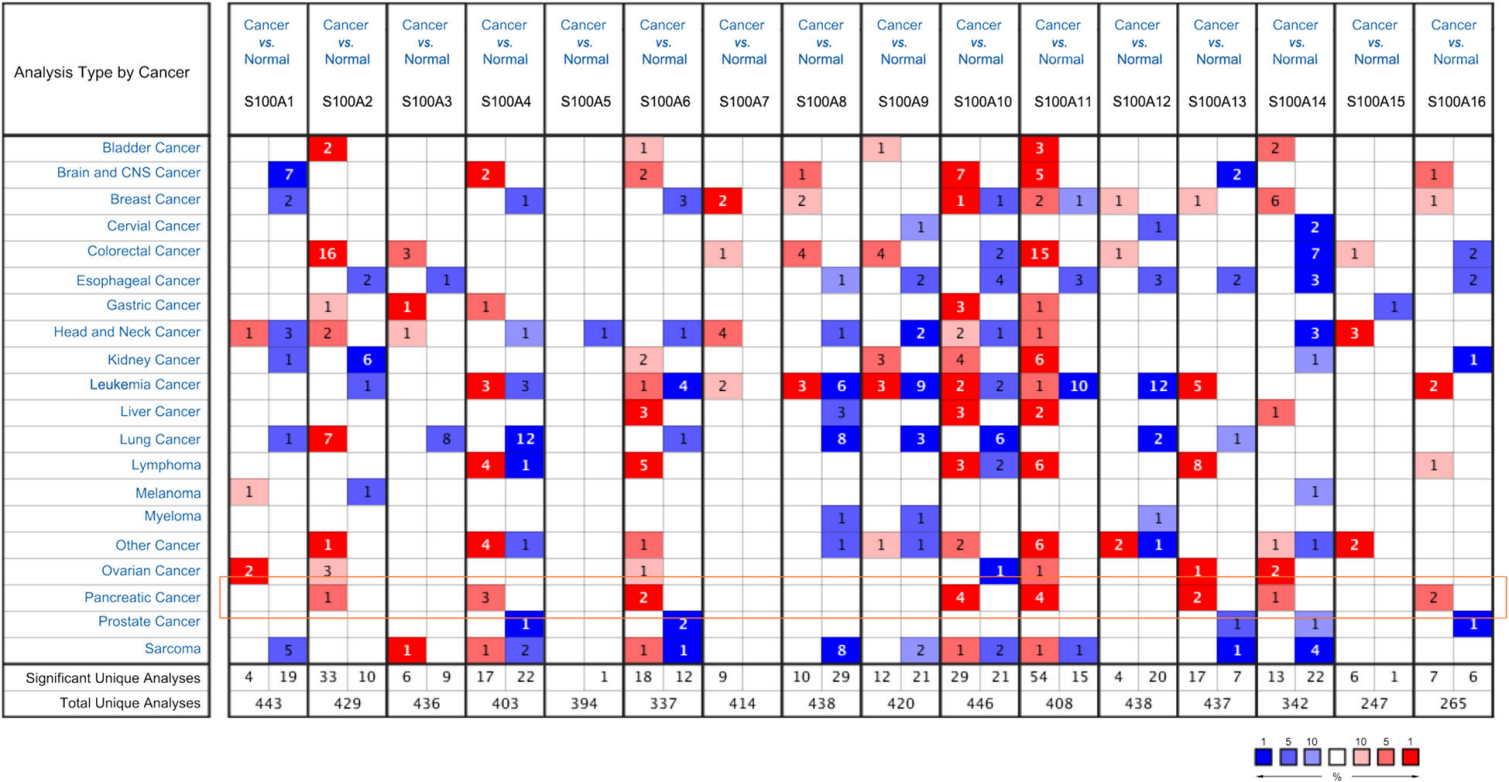
The transcription levels of S100As in various tumors (Oncomine). Difference of transcriptional expression was compared by students’ t-test. The color indicates the best gene rank percentile for the analyses. Cut-off of *p* value and fold change were as following: Data type: mRNA, p value: 1E-4, fold change: 2, gene rank: 10%

Abnormal expression of genes in tumors is often the driving factor for tumor progression. In order to explore abnormal gene expression in PDAC patients, Oncomine database was used to analyze expression levels of S100As in cancer and normal samples. We found that multiple members of the S100A family (S100A2, S100A4, S100A6, S100A10, S100A11, S100A13, S100A14, and S100A16) are highly expressed in PDAC (Fig. 1).

**Fig. 2:**
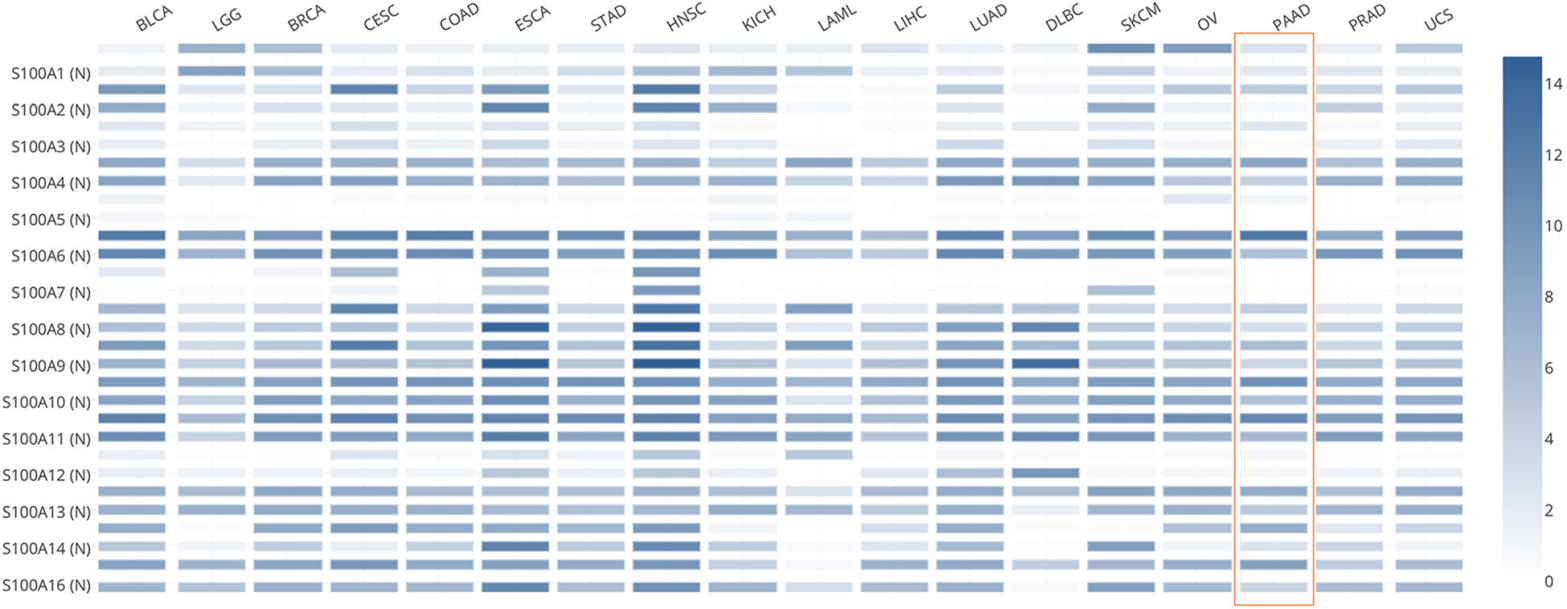
The transcription levels of S100As in various tumors (GEPIA 2). The density of color in each block represents the median expression value of a gene in a given tissue, normalized by the maximum median expression value across all blocks. Different genes in same tumors or normal tissues can be compared in one plot. PDAC, Pancreatic ductal adenocarcinoma; BLCA, Bladder Urothelial Carcinoma; LGG, Brain Lower Grade Glioma; BRCA, Breast invasive carcinoma; CESC, Cervical squamous cell carcinoma and endocervical adenocarcinoma; COAD, Colon adenocarcinoma; ESCA, Esophageal carcinoma; STAD, Stomach adenocarcinoma; HNSC, Head and Neck squamous cell carcinoma; KICH, Kidney Chromophobe; LAML, Acute Myeloid Leukemia; LIHC, Liver hepatocellular carcinoma; LUAD, Lung adenocarcinoma; DLBC, Lymphoid Neoplasm Diffuse Large B-cell Lymphoma; SKCM, Skin Cutaneous Melanoma; OV, Ovarian serous cystadenocarcinoma; PAAD, Pancreatic adenocarcinoma; PRAD, Prostate adenocarcinoma; UCS, Uterine Carcinosarcoma

**Fig. 3:**
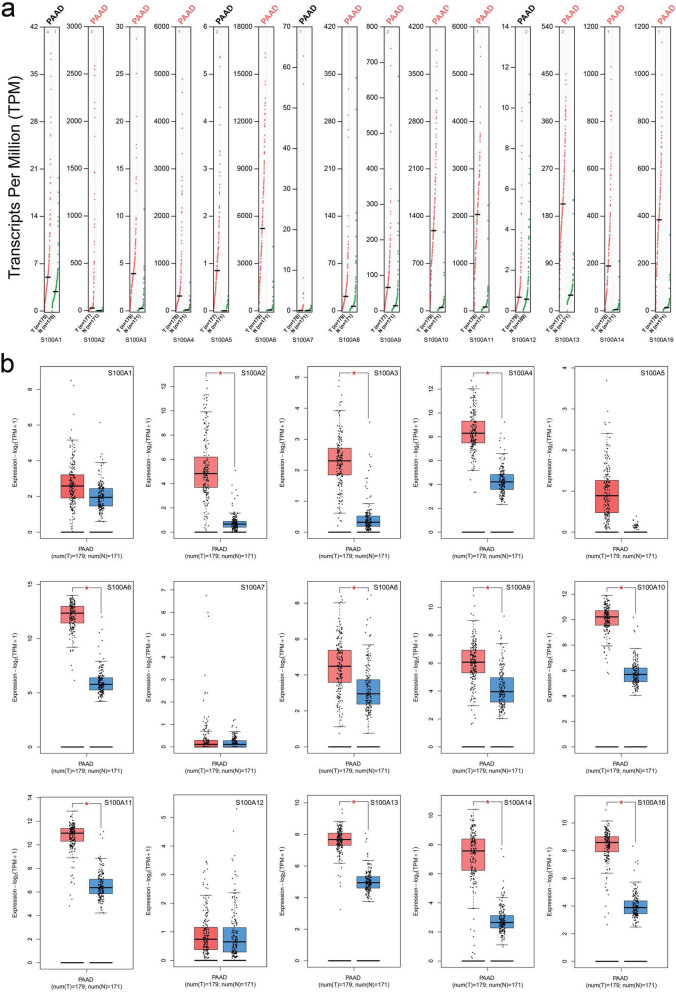
The expression of S100As in PDAC (GEPIA2). The expression of S100As in PDAC (a, scatter diagram; b, box plot).*P < 0.05. T: pancreatic tumor tissue; N: Normal pancreatic tissue

**Fig. 4:**
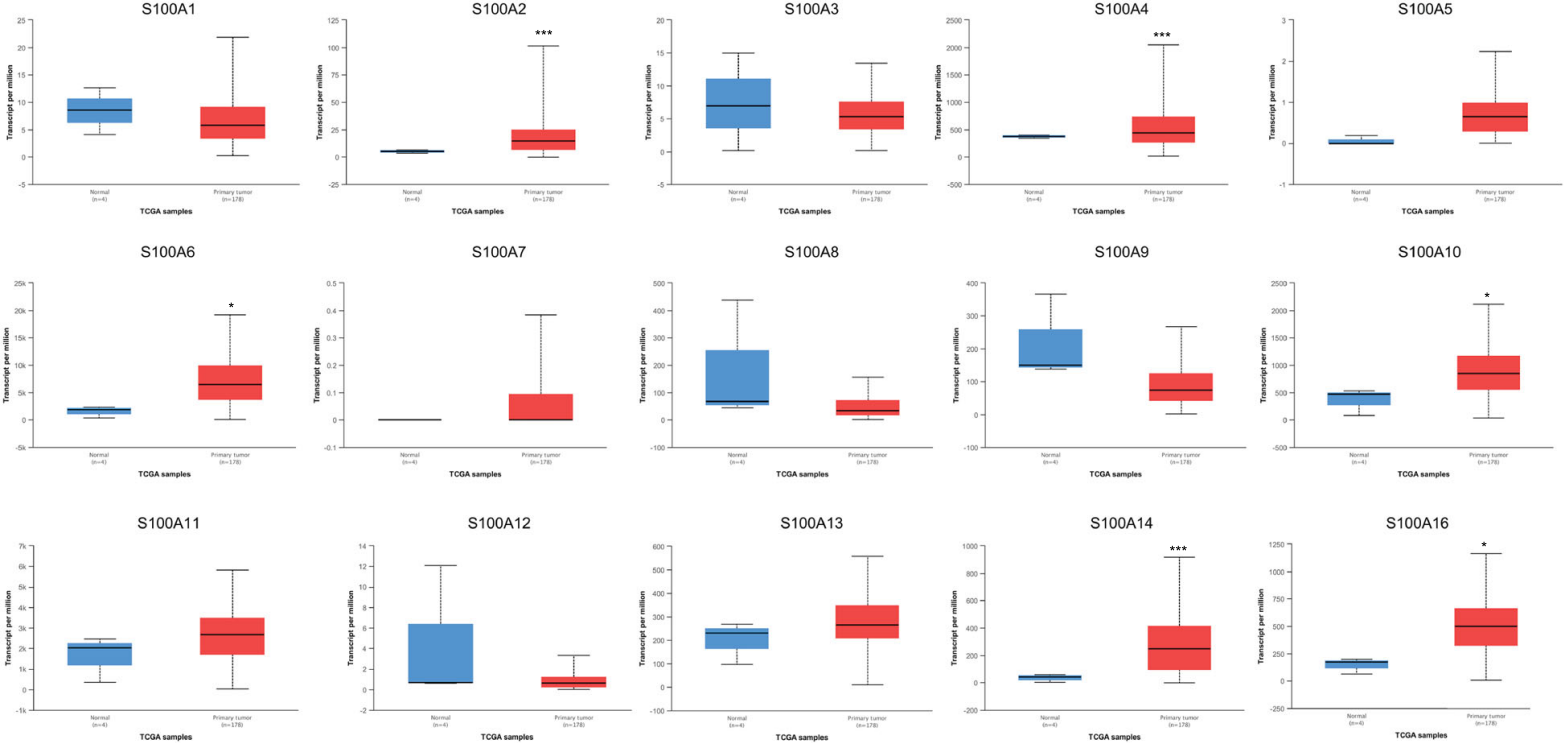
The expression of S100As in PDAC (UALCAN). Normal pancreatic tissue (*n* = 4), pancreatic cancer tissue (*n* = 178). *P < 0.05, **P < 0.01

Next, GEPIA 2 was used to obtain the expression matrix plots based on S100As family members. The color density indicates the median expression of one gene in specified tissue, and is further normalized by the maximum median expression value in all blocks. As shown in Figs. 2 and 3, significantly higher mRNA expressions of S100A2/3/4/6/8/9/10/11/13/14/16 were found in PDAC versus normal pancreatic tissue. Moreover, the transcription expression pattern of S100As family members were further analyzed by UALCAN whose resources were based on publicly available cancer OMICS data (TCGA and MET500), which was different from Oncomine database, and the results indicated that, S100A2/4/6/10/14/16 were over-expressed in PDAC samples compared with normal pancreas samples (Fig. 4). *P* values less than 0.05 between the indicated groups considered statistically.

### The transcription levels of S100A2/4/6/10/14/16 in PDAC cells and tissues

**Fig. 5:**
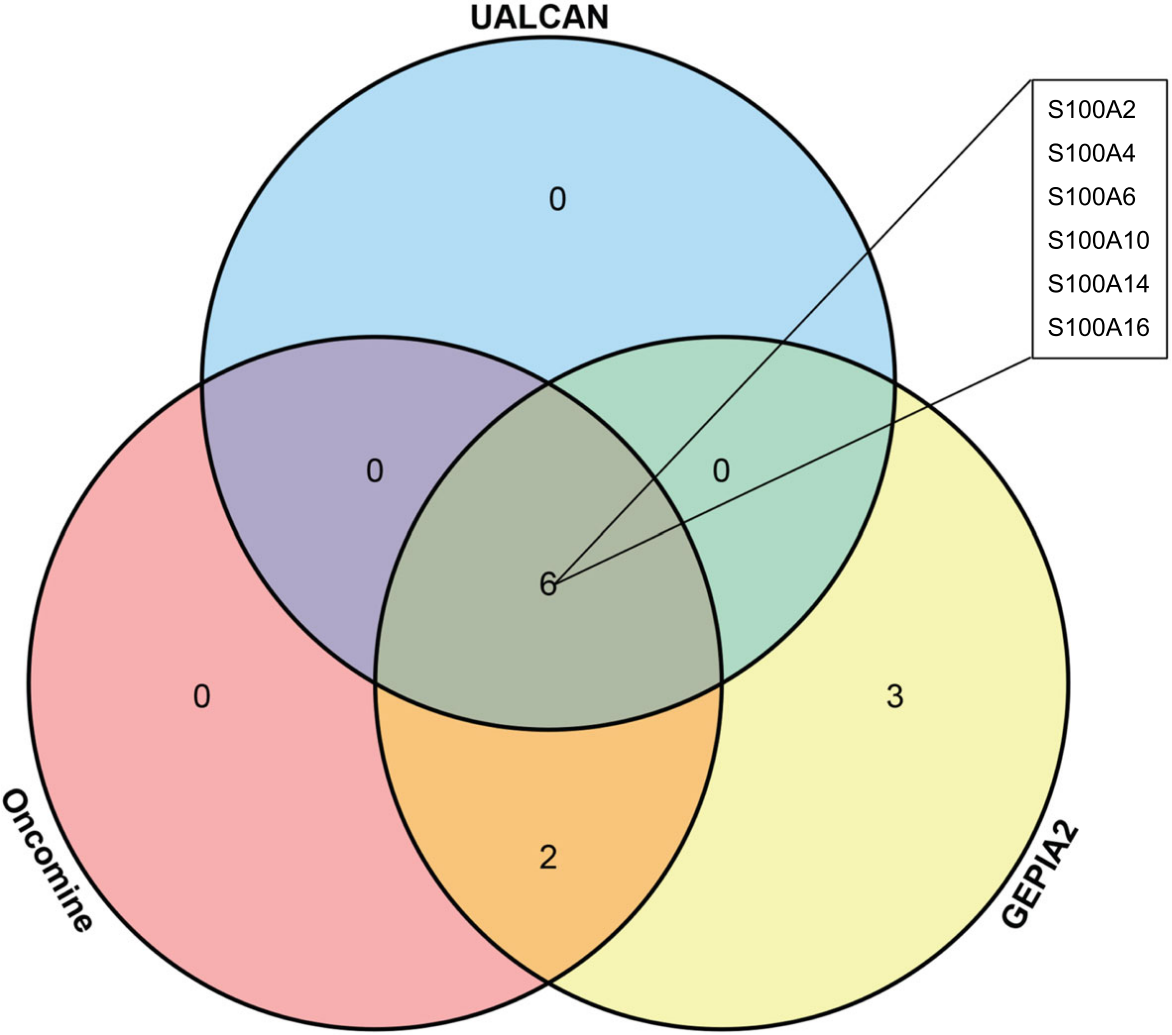
The overlap of the results in the three databases (Oncomine, GEPIA 2 and UALCAN). S100A2/4/6/10/14/16 were highly expressed in the tumor samples compared with the adjacent normal tissues

Based on the results above, we chose the overlap of the results in the three databases (Oncomine, GEPIA 2 and UALCAN), as shown in Fig. 5, S100A2, S100A4, S100A6, S100A10, S100A14 and S100A16 were over-expressed in PDAC versus normal samples. After completing the analysis based on bioinformatics, we further tried to explore the expression patterns of S100As in pancreatic cancer cell lines and pancreatic cancer tissue.

**Fig. 6:**
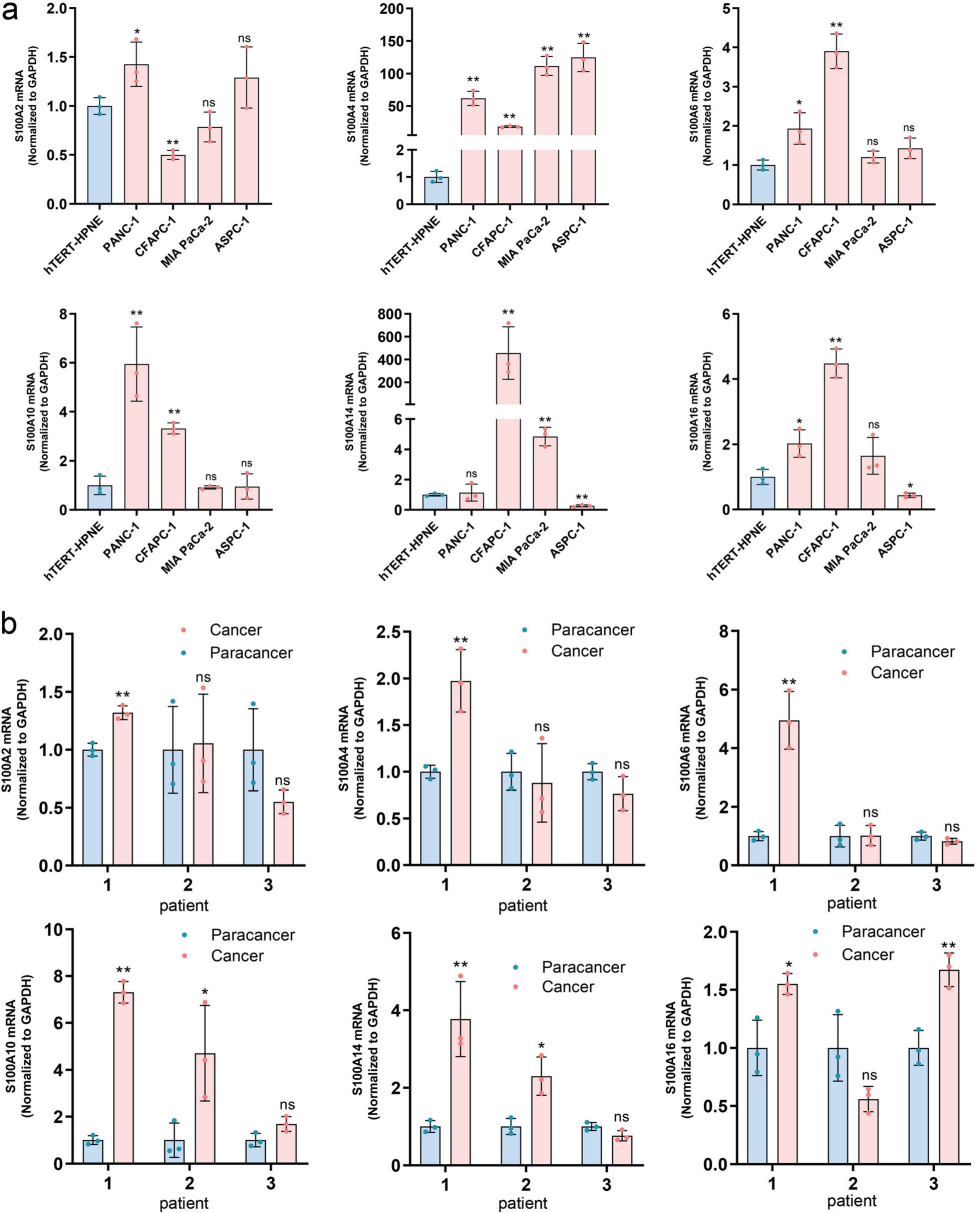
The transcription levels of S100A2/4/6/10/14/16 in PDAC cells and tissues. **a** the expression of S100A2/4/6/10/14/16 in human pancreatic cancer lines (PANC-1, CFAPC-1, MIA PaCa-2 and ASPC-1), and human pancreatic cell line hTERT-HPNE were measured by real-time PCR. **b** the expression of S100A2/4/6/10/14/16 in pancreatic cancer tissues, which were from three PDAC patients undergoing surgery, were measured by real-time PCR. *P < 0.05, **P < 0.01, ns, not significant

**Fig. 7:**
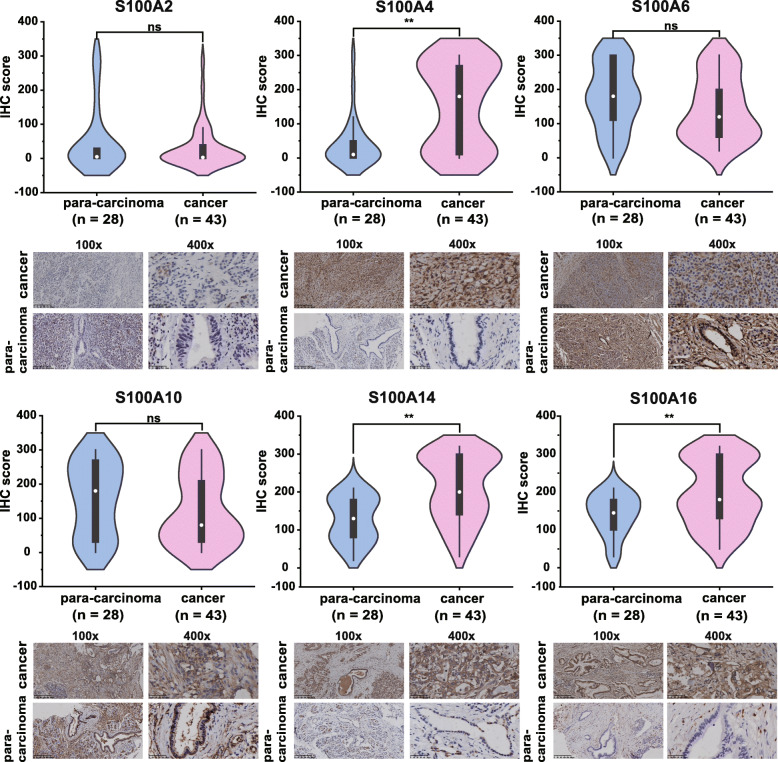
Immunohistochemical detection of S100As expression in pancreatic cancer. IHC was performed to test S100As protein level in pancreatic cancer tissues (*n* = 43) and their counterparts (*n* = 28). The intensity was scored as “zero,” “one,” “two,” and “three”; that was negative, weak, moderate, and strong staining, respectively. The positively stained cells percentage was scored as “0 to 100%” The final IHC score was obtained by product between intensity score and stained area. *P < 0.05, **P < 0.01, ns, not significant

We first analyzed the expression of S100A2/4/6/10/14/16 in human pancreatic cancer lines (PANC-1, CFAPC-1, MIA PaCa-2 and ASPC-1), and human pancreatic cell line hTERT-HPNE served as control by real-time PCR. As shown in Fig. 6a (*P < 0.05, **P < 0.01, ns *P* > 0.05), the expression level of S100A2 was higher in PANC-1 than that in hTERT-HPNE. S100A4 was up-regulated in all four pancreatic cancer cells relative to hTERT-HPNE cells. S100A6 and S100A10 were up-regulated in PANC-1, CFAPC-1 cells compared with normal control. S100A14 mRNA expression was up-regulated in CFAPC-1, MIA PaCa-2 cells and down-regulated in ASPC-1 compared to normal cells. Compared with hTERT-HPNE cells, the mRNA expression of S100A16 was significantly up-regulated in PANC-1, CFAPC-1 cells, and down-regulated in ASP-1 cells. We then tested the expression of S100A2/4/6/10/14/16 in pancreatic cancer tissues, which were from three PDAC patients undergoing surgery. From the results, we noted high expression of S100A2/4/6/10/14/16 in the pancreatic cancer tissue of patient one. However, in pancreatic cancer tissue of patient 2, only S10010 and S100A14 are highly expressed compared with adjacent cancer (Fig. 6b). Next, IHC was used to measure S100As protein level in pancreatic cancer tissues (*n* = 43) and their counterparts (*n* = 28). The data showed that S100A4, S100A14 and S100A16 were significantly higher in PDAC tissues compared with their counterparts, but the protein level of S100A2, S100A6 and S100A10 were similar between cancer tissues and their counterparts (Fig. 7).

### Analysis of S100As methylation levels

**Fig. 8:**
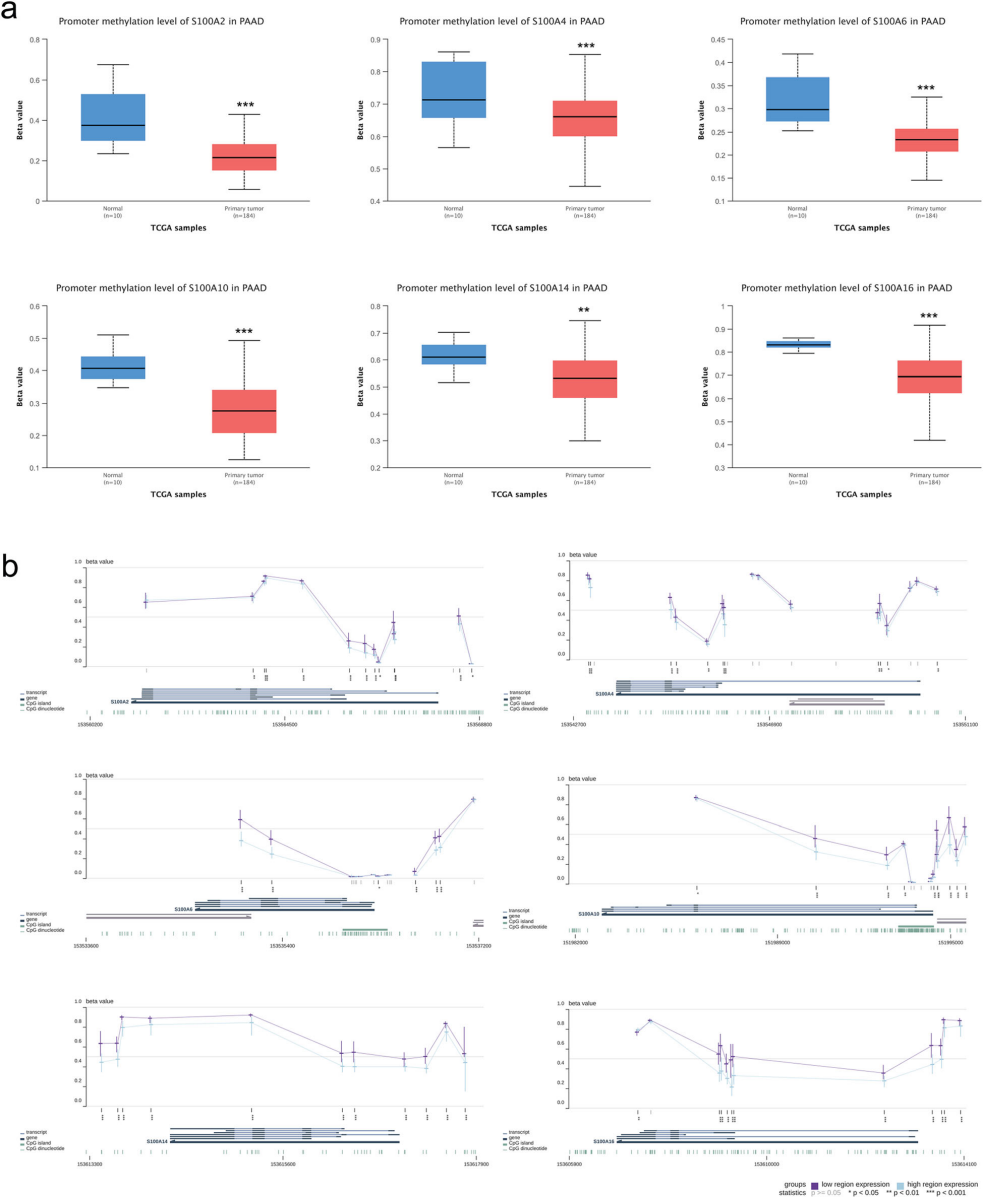
Analysis of S100As methylation levels in PDAC. **a** promoter methylation level of S100As were detected by UALCAN database. **P < 0.01, ***P < 0.001. **b** specific methylation sites were analyzed by MEXPRESS database (blue indicates a low level of expression, while violet indicates a high level of expression)

The level of DNA methylation is inversely proportional to transcriptional activity and affects genome stability. Additionally, the methylation levels of the promoter region have a greater effect on gene expression activity. We first measured the promoter methylation level of the S100A2/4/6/10/14/16 in PDAC by searching the UALCAN database. The results showed that (Fig. 8a), compared with normal tissues, the promoter methylation level of S100A2, S100A4, S100A6, S100A10, S100A14, S100A16 in PDAC (*n* = 10) were lower compared with normal tissue (*n* = 184), **P < 0.01, ***P < 0.001. We further used the MEXPRESS database to analyze specific methylation sites. According to MEXPRESS’s algorithm, PDAC tissue samples were divided into two groups according to their S100As expression level (blue indicates a low level of expression, while violet indicates a high level of expression). The horizontal line at each probe position represents the median percentage of methylation (β value), while the vertical line represents the range between 25 and 75%. As shown in Fig. 8b, in PDAC tissues, the expression of S100A2/4/6/10/14/16 were negative correlation (*P < 0.05, **P < 0.01, ***P < 0.001) to the level of promoter methylation.

### Correlation analysis of mRNA expression of S100As and clinicopathological parameters in pancreatic cancer

**Fig. 9:**
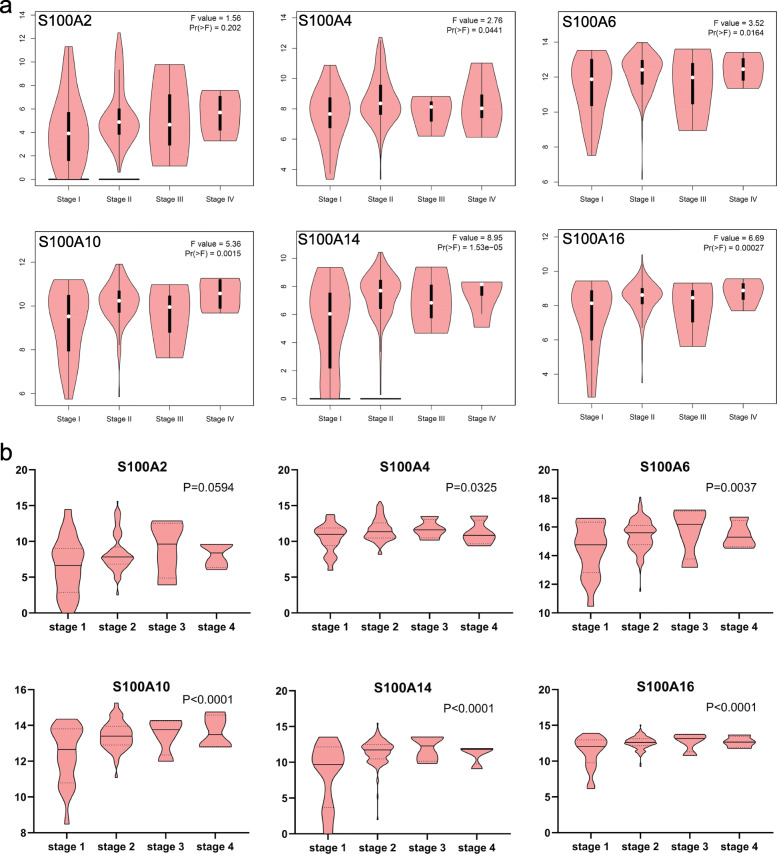
Correlation between S100As expression and tumor stage in PDAC. Correlation between S100As expression and tumor stage in PDAC (**a** GEPIA2; **b** Linkedomics database). *P* < 0.05 was considered to be statistically significant

After finding that the transcriptional level of S100A2/4/6/10/14/16 was highly expressed in pancreatic cancer, we further analyzed the expression of S100A2/4/6/10/14/16 with tumor stage for pancreatic cancer using GEPIA2 and Linkedomics database. As shown in Fig. 9a, S100A4/6/10/14/16 groups significantly varied, whereas S100A2 did not significantly (*P* values less than 0.05 was considered to be statistically significant). The results of Linkedomics database analysis from different sample sources are consistent with those of GEPIA2 (Fig. 9b). In short, we could found from the results that, mRNA expressions of S100A4/6/10/14/16 were significantly associated with tumor grades, which may have potential value of clinical transformation.

### Prognostic value analysis of S100As mRNA expression in pancreatic cancer

**Fig. 10:**
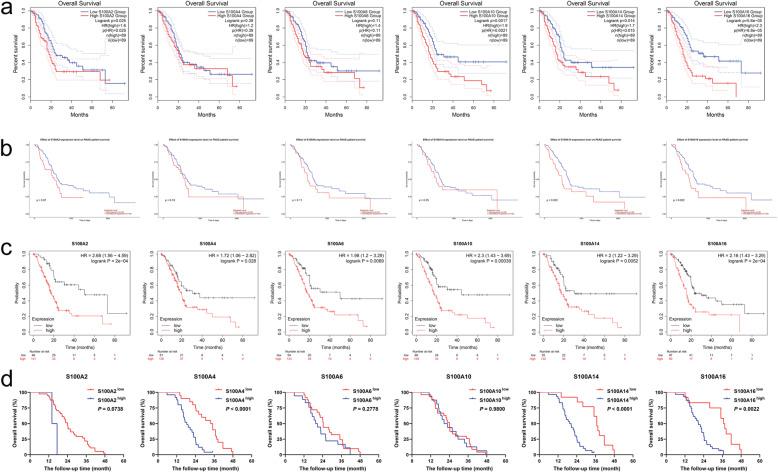
Prognostic value of mRNA expression of S100As in PDAC patients. The expression and prognosis analysis of S100A2/4/6/10/14/16 in PDAC patients according to **a** GEPIA 2; **b** UALCAN; **c** Kaplan-Meier Plotter. **d** the prognostic value of S100A2/4/6/10/14/16 were evaluated in 43 cases of PDAC patients. *P* < 0.05 was considered to be statistically significant

In order to explore the critical efficiency of S100A2/4/6/10/14/16 in the survival of patients with pancreatic cancer, GEPIA2, UALCAN and Kaplan-Meier Plotter tools, whose data sources include GEO, EGA, and TCGA, were used to analyze the prognostic values (OS) of the mRNA expression of S100As in pancreatic cancer patients. Based on the high expression of S100As members in pancreatic cancer, the association between mRNA expression of S100A2/4/6/10/14/16 and prognosis of pancreatic cancer patients were further analyzed. As shown in Fig. 10a, the results of the GEPIA2 database indicate that, higher mRNA expression of S100A2/10/14/16 were significantly associated with shorter OS in PDAC patients (*P* < 0.05). The results analyzed by the UALCAN database dedicated that (Fig. 10b), expression levels of S100A14 and S100A16 were significantly correlated with the OS of PDAC patients (*P < 0.05). In addition, Kaplan-Meier plotter were used to analyze the prognostic values of S100A2/4/6/10/14/16 in PDAC. As shown in Fig. 10c, mRNA expression of S100A2/4/6/10/14/16 were inversely correlated with patient survival (*P* < 0.01).

Next, we further evaluated the prognostic value of S100A2/4/6/10/14/16 in 43 cases of PDAC patients. It was revealed from Kaplan-Meier analyses that the S100A4, S100A14 and S100A16-high patients have worse overall survival rate than S100A4, S100A14 and S100A16-low patients. However, there are no differences of overall survival rate between patients who have different protein level of S100A2, S100A6 and S100A10 (Fig. 10d). The correlation between S100A2, S100A4, S100A6, S100A10, S100A14 and S100A16 level and clinicopathologic characteristics were shown in supplementary Tables S3–8, respectively.

### Correlation analysis of drugs targeting S100As

To explore the potential use of S100As as host targets, we queried DrugBank (version 5.1.6) to identify drugs targeting them. As shown in Table 1, there are seven kinds of drugs targeting S100A2, which can be further divided into antagonist, inactivator, and regulator groups. There are two drugs (Trifluoperazine and Copper) targeting S100A4. In addition, three drugs that target S100A6 have been approved. Three drugs with actions of ligand were identified as drugs targeting S100A16.While, drugs targeting S100A10 and S100A14 have not yet been found now.

**Table 1:** Drug relations of S100As

Gene	Drug relations				
S100A2	Drugbank ID	Drug Name	Drug Group	Actions	Reference
	DB00768	Olopatadine	approved	Antagonist	[15]
	DB01373	Calcium	nutraceutical		[16]
	DB01593	Zinc	approved, investigational		[16]
	DB09130	Copper	approved, investigational		[17]
	DB14487	Zinc acetate	approved, investigational		[16]
	DB14533	Zinc chloride	approved, investigational	Inactivator, regulator	[16]
	DB14548	Zinc sulfate, unspecified form	approved, experimental	Inactivator, regulator	[16]
S100A4	DB00831	Trifluoperazine	approved, investigational	Inhibitor	[18]
	DB09130	Copper	approved, investigational		[17]
S100A6	DB11093	Calcium citrate	approved, investigational	Ligand	UniProt: Protein S100-A6
	DB11348	Calcium Phosphate	approved	Ligand	UniProt: Protein S100-A6
	DB14481	Calcium phosphate dihydrate	approved		UniProt: Protein S100-A6
S100A10	NA				
S100A14	NA				
S100A16	DB11093	Calcium citrate	approved, investigational	Ligand	UniProt: Protein S100-A16
	DB11348	Calcium Phosphate	approved	Ligand	UniProt: Protein S100-A16
	DB14481	Calcium phosphate dihydrate	approved		UniProt: Protein S100-A16

### Genetic mutations in S100As and their associations with OS and (progression-free survival) (PFS) of PDAC patients

**Fig. 11:**
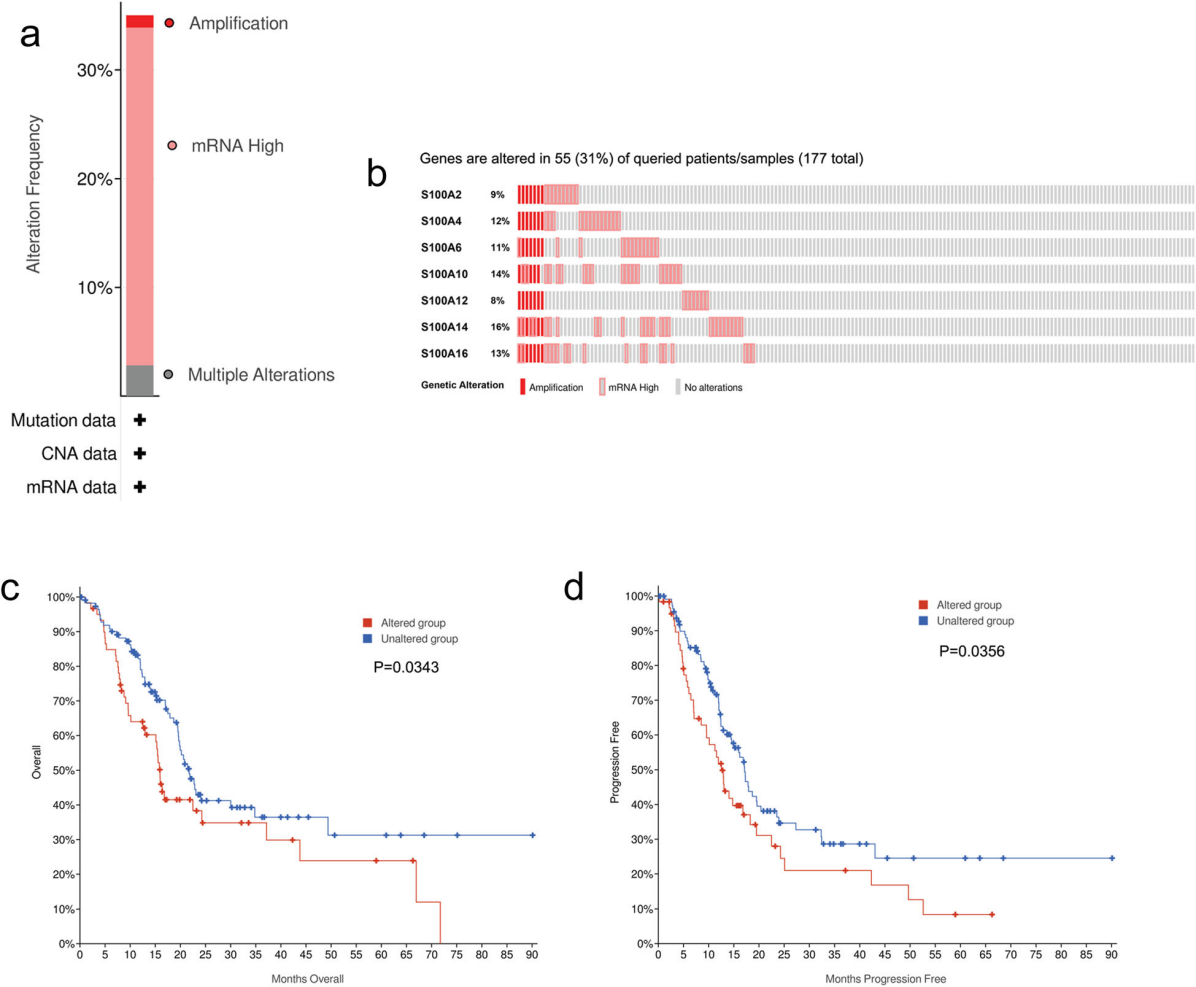
Genetic mutations in S100As and their association with OS and PFS of PDAC patients (cBioPortal). High mutation rate (31%) of S100As was observed in PDAC patients. S100A14, S100A10 and S100A16 ranked the highest three genes of genetic alterations, and their mutation rates were 16, 14 and 13%, respectively. **a**, S100As expression and mutation analysis in PDAC. **b**-**c**, genetic alterations in S100As were associated with shorter OS and PFS of PDAC patients

Since gene mutation is an important factor affecting its expression, we further analyzed the genetic alteration in S100A2/4/6/10/14/16 and their correlation with OS and PFS in patients with pancreatic cancer by using cBioPortal online tool. As shown in Fig. 11a-b, S100A2/4/6/10/14/16 were altered in 55 samples of 177 patients with pancreatic cancer (31%). The mutation rates of S100A2, S100A4, S100A6, S100A10, S100A14 and S100A16 were 9, 12, 11, 14, 8, 16 and 13%, respectively. In additional, results from Kaplan-Meier plot and log-rank test indicated that genetic alteration in S100A2/4/6/10/14/16 were associated with shorter OS (Fig. 11c, *p* = 0.0343) and PFS (Fig. 11d, *p* = 0.0356) of PDAC patients. Survival analysis showed that gene mutation of S100As might also significantly affect the prognosis of PDAC patients.

### Predicted functions and pathways of the genes that positively correlated to S100As in PDAC patients

**Fig. 12:**
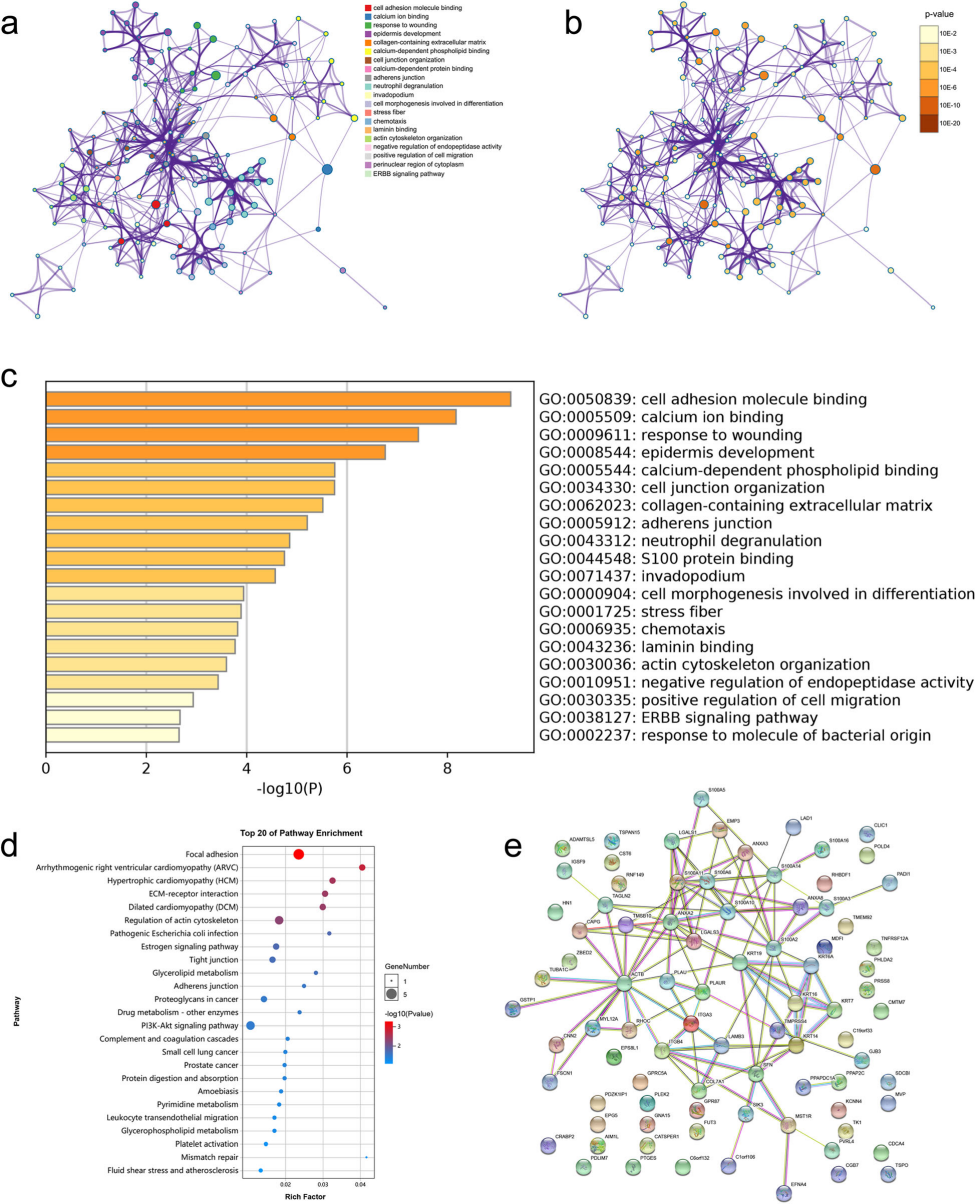
Protein-protein interaction enrichment analysis. Predicted functions and pathways of significant genes positively correlated to S100As in pancreatic cancer. Network of enriched terms: **a** enriched ontology clusters colored by cluster ID (legend on right), where nodes that share the same cluster ID are typically close to each other; **b** colored by *p*-value, where terms containing more genes tend to have a more significant p-value. **c** Bar graph of enriched terms across significant genes positively correlated to S100As, colored by *p*-values. **d** top 20 of pathway enrichment of genes positively correlated to S100As. **e** information of interactions of genes positively correlated to S100As were extracted from online database STRING

Linkedomics database was used to search for significant genes positively correlated to S100As in pancreatic cancer samples. As shown in Table S2, we selected top25 genes, which were positively related to expression pattern of S100As, according to the person correlation. Furthermore, the positively correlated significant genes were loaded into Metascape to acquire gene ontology (GO) enrichment. As shown in Fig. 12a-b, the enriched ontology clusters colored by cluster ID, which predicted the functional roles of S100As based on molecular functions, cellular components and biological processes. We could found that GO:0050839 (cell adhesion molecule binding), GO:0005509 (calcium ion binding), GO:0009611 (response to wounding), GO:0008544 (epidermis development) were significantly regulated (top 4) by the positively related to expression pattern of S100As in pancreatic cancer (Fig. 12c). In addition, KEGG pathways enrichment analysis was performed by the online free tool provided by omicshare. The heatmap are colored by their *P*-values (Fig. 12d). The result suggested that, focal adhesion; arrhythmogenic right ventricular cardiomyopathy and hypertrophic cardiomyopathy were significantly associated with the genes which were positively correlated significant Genes of S100As. STRING, a database that can analyze the interaction between known proteins and predicted proteins, was used to predict protein-protein interactions of the genes positively related to S100As expression described above. We can see from Fig. 12e, S100As family members have close interactions themselves. In addition, they also interact with tumor cell adhesion-related proteins such as ANXA2, LGALS3, ANXA8, and ACTB.

## Discussion

PDAC remains a treatment-refractory malignancy with poor prognosis. The discovery of some promising molecular targets for the treatment of PDAC provided a new direction for the diagnosis and treatment of PDAC. For example, Jin et al. reported that, PES1 might be a promoting factor of tumor growth and a PDAC prognosis associated protein [19]. Goehrig et al. found that, βig-h3 stromal-derived protein is a main factor driving the immune paracrine interaction mechanism of PDAC, which might be novel target in PDAC treatment [20]. However, compared with other malignant tumors, effective diagnosis and treatment targets of pancreatic cancer are still quite lacking. It is urgent to develop screening procedures for early detection and more efficacious treatment strategies for PDAC [21]. Bioinformatics provides an opportunity to quest for the genetic alterations in pancreatic cancer, and proved a useful way to identify new biomarkers in pancreatic cancer. To verify the results from bioinformatics further, we tested the expression of S100As in pancreatic cancer at the cellular and tissue levels by real-time PCR and immunohistochemistry. Unfortunately, pancreatic cancer tissues from PDAC patients undergoing surgery are scarce due to the few cases of pancreatic cancer surgery. Because the sample size is too small, the heterogeneity between patients obscures some of the original expression levels. Here, we investigated the mRNA expression and prognostic values of S100A family in PDAC for the first time. We expect that our findings could help us to understand the mechanism of PDCA, improve the clinical treatment design, and improve the reliability of prognostic assessment.

The S100 protein family belong to a family of calcium binding proteins (CaBPs) and contain 25 known members, which have a high degree of sequence and structural similarity. The S100A subfamily is among the most distinctive of EF-hand CaBPs and only found in vertebrates. Studies have shown that multiple members of the S100As are abnormally expressed in tumors and are widely involved in processes related to tumorigenesis and progression [22, 23]. However, the function of most S100A has not been fully characterized, especially in pancreatic cancer, the research on S100A is still very limited.

**Fig. 13:**
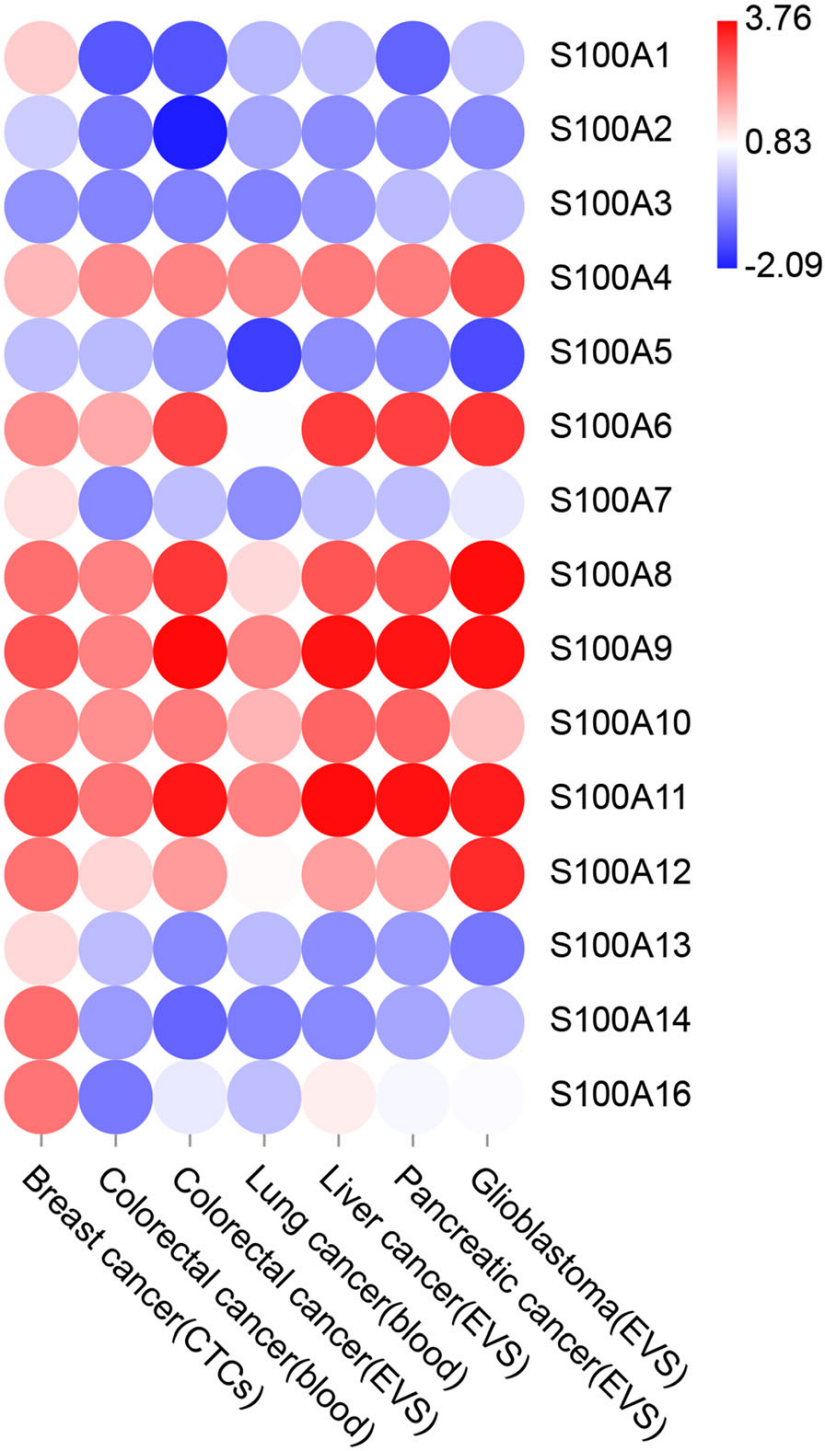
Potential clinical significance of RNAs as biomarkers. BBCancer (http://bbcancer.renlab.org/), a database that provides potential clinical significance of RNAs as biomarkers for early cancer detection, was used to analyze the value of S100A in the early diagnosis of PDAC

Among the S100As, S100A4 is the most intensively studied. S100A4 is a member of the S100As, which contains two EF-hand calcium-binding motifs. Among its related pathways are Ca^2+^, cAMP and Lipid Signaling. Fei et al. reported that, the expression of S100A4 was positively correlated with the malignancy degree and the level in lymph node metastasis of human colorectal cancer [24]. Recently, a study about hepatocellular carcinogenesis indicated that, high expression of S100A4 in the fibrotic region of was liver tumor tissue observed, and the expression of S100A4 was associated with advanced disease severity [25]. It was also reported that, S100A4 was one of the key factors in EMT mediated by Shh-Gli1 signaling pathway in PDAC [26]. In present study, Oncomine, GEPIA 2 and UALCAN datasets revealed that the expression of S100A4 was higher in PDAC than in normal pancreatic tissues. We also noticed that, S100A4 was up-regulated in all four pancreatic cancer cells relative to hTERT-HPNE cells. In addition, BBCancer (http://bbcancer.renlab.org/), a database that provides potential clinical significance of RNAs as biomarkers for early cancer detection, was used to analyze the value of S100A in the early diagnosis of pancreatic cancer. We can see from Fig. 13, S100A4/6/8/9/10/11/12/16 were up regulated in extracellular vesicles of pancreatic cancer, which might have diagnostic value.

As an important paralog of S100A4, S100A2 is reported to be abnormally highly expressed in various tumors, such as colorectal cancer [27] and lung cancer [28]. In our study, we indicated that the expression of S100A2 in pancreatic cancer tissues was higher than that in normal pancreatic tissues. It has also been suggested as a negative prognostic biomarker in pancreatic cancer [29]. However, the prognostic role of S100A2 in pancreatic cancer remains to be studied. GO annotations of S100A2 include calcium ion binding and identical protein binding. Among its related pathways are p53 pathway.

S100A6 is also an up-regulated S100As in pancreatic cancer confirmed by bioinformatics and in vitro experiments. Results of BBCancer also showed that, S100A6 was up-regulated in extracellular vesicles of PDAC. Diseases associated with S100A6 include Pilomatrixoma and Retinitis Pigmentosa. The latest research also showed that S100A6 is involved in the process of neurodegenerative diseases [30]. GO annotations of S100A6 were calcium ion binding and calcium-dependent protein binding. Among its related pathways are prostaglandin synthesis and regulation and DNA damage. Further research is needed to explain the role of S100A6 in the development of pancreatic cancer.

Diseases associated with S100A10 include Trachea Leiomyoma and Barrett’s Adenocarcinoma. There is evidence that S100A10 has potential value as a biomarker that represents the high-grade cell state of breast cancer [31]. Among its related pathways are Prostaglandin Synthesis and Regulation and Response to elevated platelet cytosolic Ca^2+^. GO annotations related to this gene include calcium ion binding and lipid binding. An important paralog of this gene are S100A1and S100A14. In our study, both bioinformatics analysis and experimental verification indicated that S100A10 was up-regulated in pancreatic cancer (vs normal controls/paraneoplastic tissue). Low expression of S100A10 was associated significantly with better survival in pancreatic cancer by univariate analysis (*P* < 0.05). The above results reveal the potential value of S100A10 as a diagnosis and treatment of pancreatic cancer.

Both S100A14 and S100A16 were up-regulated in pancreatic cancer, which was also verified in our experiments. S100A14 has a potential to be clinically useful as prognostic biomarker in several cancer types. For example, Meta-analysis form Hu et al. suggested that S100A14 overexpression might be a predictive biomarker for poor prognosis in patients with breast cancer and ovarian cancer [32]. However, the role of S100A14 in pancreatic cancer is rarely reported. Survival analysis showed that, low expression of S100A14 was associated with significantly better survival. S100A16 was discovered late and its research is not deep enough. In this study, we found that S100A16 is upregulated in PDAC, and its low expression was closely related to a better survival.

Given that the signaling pathways involved in calcium ions have been shown to change the sensitivity of chemotherapy drugs [33]. We therefore also analyzed the drug relations of S100As by Drugbank. As listed in Table 1, Drugs targeted at S100As include Olopatadine, Trifluoperazine and calcium citrate. These drugs are mainly used as inactivators, regulators, corrosion inhibitors and ligands to play the role of drugs. Therefore, S100As have certain potential value in the development of pancreatic cancer chemotherapy resistance and new drug development.

DNA methylation generally occurs at the CpG site, and the conversion of cytosine to 5-methylcytosine is catalyzed by DNA methyltransferase in human genes. Approximately 80% of CpG sites are methylated, and the level of DNA methylation is inversely proportional to transcriptional activity and affects genome stability. Compared with the methylation of the gene-coding region, the methylation of the promoter region has a greater effect on gene expression activity. In our study, we found that the methylation level of the promoter region was negatively correlated with its expression, which may be a reason for the abnormal expression of S100As in pancreatic cancer.

Naturally, there are also many limitations in our research. First, although high mRNA expressions of S100A2/4/6/10/14/16 were independent prognostic factors for shorter OS of pancreatic cancer patients, the online databases used in this study lacked proteome-level data. Further studies consist of larger sample sizes (more clinical samples and different pancreatic cancer cell lines) are required to validate the findings above and to explore the clinical application of S100A2/4/6/10/14/16 in the treatment of PDAC. In addition, we did not explore the potential molecular mechanism of S100As in the occurrence and development of pancreatic cancer. Future research worth to investigate the detailed mechanism between S100As and PDAC.

## Conclusions

In summary, we comprehensively explored the expression pattern and prognostic value of S100As in PDAC, and provided an understanding of the molecular biological properties of PDAC. The current research results indicate that the increased expression of S100A2/4/6/10/14/16 in PDAC tissue may play an important role in the occurrence of pancreatic cancer. The high expression of S100A2/10/14/16 could also be used as molecular markers to identify high-risk subgroups of PDAC patients. S100A2/10/14/16 were potential therapeutic targets for PDAC, and regulating the expression of S100A2/4/6/10/14/16 might be potential prognostic markers to improve PDAC survival rate and prognostic accuracy. It is undeniable that cancer biomarkers prediction and analysis based on bioinformatics can only provide preliminary data, whose clinical applications are yet to be further developed. Further experimental verification and molecular mechanism research are also the focus of our future work.

## Supplementary Information

**Additional file 1: Supplementary Table S1.** Primers used in this study.

**Additional file 2: Supplementary Table S2.** Positively Correlated Significant Genes.

**Additional file 3: Supplementary Table S3–8**. Basic characteristics of 43 PDAC patients.

## Data Availability

The datasets presented in this study are available in online repositories.

## Abbreviations

PDAC: Pancreatic ductal adenocarcinoma
CaBPs: Calcium binding proteins
GO: Gene ontology
IHC: Immunohistochemistry
OS: Overall survival
