# Supplementary material for: Comprehensive analysis of the transcriptional expressions and prognostic value of S100A family in pancreatic ductal adenocarcinoma
Source: BMC Cancer. 2021 Sep 16;21:1039. doi: 10.1186/s12885-021-08769-6 (PMC8447682; doi:10.1186/s12885-021-08769-6)
Supplement: Supplementary file 1 — Additional file 1: Supplementary Table S1. Primers used in this study. [file 12885_2021_8769_MOESM1_ESM.docx]

Table S1 Primers used in this study

| **Primers for Real-time PCR** |  |
| --- | --- |
| S100A2 Forward | GCCAAGAGGGCGACAAGTT |
| S100A2 Reverse | AGGAAAACAGCATACTCCTGGA |
| S100A4 Forward | GATGAGCAACTTGGACAGCAA |
| S100A4 Reverse | CTGGGCTGCTTATCTGGGAAG |
| S100A6 Forward | GGGAGGGTGACAAGCACAC |
| S100A6 Reverse | AGCTTCGAGCCAATGGTGAG |
| S100A10 Forward | GGCTACTTAACAAAGGAGGACC |
| S100A10 Reverse | GAGGCCCGCAATTAGGGAAA |
| S100A14 Forward | GAGACGCTGACCCCTTCTG |
| S100A14 Reverse | CTTGGCCGCTTCTCCAATCA |
| S100A16 Forward | ATGTCAGACTGCTACACGGAG |
| S100A16 Reverse | GTTCTTGACCAGGCTGTACTTAG |
| GAPDH Forward | GTCAAGGCTGAGAACGGGAA |
| GAPDH Reverse | AAATGAGCCCCAGCCTTCTC |
