# Supplementary material for: Comprehensive analysis of the transcriptional expressions and prognostic value of S100A family in pancreatic ductal adenocarcinoma
Source: BMC Cancer. 2021 Sep 16;21:1039. doi: 10.1186/s12885-021-08769-6 (PMC8447682; doi:10.1186/s12885-021-08769-6)
Supplement: Supplementary file 2 — Additional file 2: Supplementary Table S2. Positively Correlated Significant Genes. [file 12885_2021_8769_MOESM2_ESM.docx]

Table S2 Positively Correlated Significant Genes

| **Select** | **Target Gene/Attribute** | **Person Correlation** | **P-value^1^** | **FDR (BH)^2^** | **Event_SD^3^** | **Event_TD^4^** |
| --- | --- | --- | --- | --- | --- | --- |
| S100A2 | [S100A2](https://www.ncbi.nlm.nih.gov/gene/?term=S100A2%5bSymbol%5d%20AND%20Homo%20sapiens) | 1.000e+00 | 1.000e-28 | 1.000e-24 | 1.77e+02 | 1.77e+02 |
|  | [GPR87](https://www.ncbi.nlm.nih.gov/gene/?term=GPR87%5bSymbol%5d%20AND%20Homo%20sapiens) | 7.100e-01 | 1.315e-28 | 1.300e-24 | 1.77e+02 | 1.70e+02 |
|  | [ANXA8](https://www.ncbi.nlm.nih.gov/gene/?term=ANXA8%5bSymbol%5d%20AND%20Homo%20sapiens) | 6.936e-01 | 7.278e-27 | 4.797e-23 | 1.77e+02 | 1.74e+02 |
|  | [ITGA3](https://www.ncbi.nlm.nih.gov/gene/?term=ITGA3%5bSymbol%5d%20AND%20Homo%20sapiens) | 6.813e-01 | 1.248e-25 | 6.170e-22 | 1.77e+02 | 1.78e+02 |
|  | [ANXA8L2](https://www.ncbi.nlm.nih.gov/gene/?term=ANXA8L2%5bSymbol%5d%20AND%20Homo%20sapiens) | 6.798e-01 | 1.764e-25 | 6.975e-22 | 1.77e+02 | 1.71e+02 |
|  | [PTGES](https://www.ncbi.nlm.nih.gov/gene/?term=PTGES%5bSymbol%5d%20AND%20Homo%20sapiens) | 6.686e-01 | 2.029e-24 | 6.014e-21 | 1.77e+02 | 1.78e+02 |
|  | [CDCA4](https://www.ncbi.nlm.nih.gov/gene/?term=CDCA4%5bSymbol%5d%20AND%20Homo%20sapiens) | 6.684e-01 | 2.129e-24 | 6.014e-21 | 1.77e+02 | 1.78e+02 |
|  | [FSCN1](https://www.ncbi.nlm.nih.gov/gene/?term=FSCN1%5bSymbol%5d%20AND%20Homo%20sapiens) | 6.613e-01 | 9.548e-24 | 2.360e-20 | 1.77e+02 | 1.78e+02 |
|  | [CMTM7](https://www.ncbi.nlm.nih.gov/gene/?term=CMTM7%5bSymbol%5d%20AND%20Homo%20sapiens) | 6.594e-01 | 1.401e-23 | 3.079e-20 | 1.77e+02 | 1.78e+02 |
|  | [TK1](https://www.ncbi.nlm.nih.gov/gene/?term=TK1%5bSymbol%5d%20AND%20Homo%20sapiens) | 6.567e-01 | 2.469e-23 | 4.882e-20 | 1.77e+02 | 1.78e+02 |
|  | [COL7A1](https://www.ncbi.nlm.nih.gov/gene/?term=COL7A1%5bSymbol%5d%20AND%20Homo%20sapiens) | 6.543e-01 | 3.976e-23 | 7.148e-20 | 1.77e+02 | 1.78e+02 |
|  | [KRT6A](https://www.ncbi.nlm.nih.gov/gene/?term=KRT6A%5bSymbol%5d%20AND%20Homo%20sapiens) | 6.532e-01 | 5.042e-23 | 8.309e-20 | 1.77e+02 | 1.70e+02 |
|  | [KRT7](https://www.ncbi.nlm.nih.gov/gene/?term=KRT7%5bSymbol%5d%20AND%20Homo%20sapiens) | 6.524e-01 | 5.922e-23 | 9.008e-20 | 1.77e+02 | 1.77e+02 |
|  | [S100A11](https://www.ncbi.nlm.nih.gov/gene/?term=S100A11%5bSymbol%5d%20AND%20Homo%20sapiens) | 6.519e-01 | 6.575e-23 | 9.287e-20 | 1.77e+02 | 1.78e+02 |
|  | [GNA15](https://www.ncbi.nlm.nih.gov/gene/?term=GNA15%5bSymbol%5d%20AND%20Homo%20sapiens) | 6.509e-01 | 8.003e-23 | 9.923e-20 | 1.77e+02 | 1.78e+02 |
|  | [KRT14](https://www.ncbi.nlm.nih.gov/gene/?term=KRT14%5bSymbol%5d%20AND%20Homo%20sapiens) | 6.509e-01 | 8.029e-23 | 9.923e-20 | 1.77e+02 | 1.74e+02 |
|  | [NCRNA00152](https://www.ncbi.nlm.nih.gov/gene/?term=NCRNA00152%5bSymbol%5d%20AND%20Homo%20sapiens) | 6.489e-01 | 1.182e-22 | 1.375e-19 | 1.77e+02 | 1.78e+02 |
|  | [S100A16](https://www.ncbi.nlm.nih.gov/gene/?term=S100A16%5bSymbol%5d%20AND%20Homo%20sapiens) | 6.480e-01 | 1.432e-22 | 1.573e-19 | 1.77e+02 | 1.78e+02 |
|  | [LOC642587](https://www.ncbi.nlm.nih.gov/gene/?term=LOC642587%5bSymbol%5d%20AND%20Homo%20sapiens) | 6.446e-01 | 2.756e-22 | 2.740e-19 | 1.77e+02 | 1.23e+02 |
|  | [ANXA2](https://www.ncbi.nlm.nih.gov/gene/?term=ANXA2%5bSymbol%5d%20AND%20Homo%20sapiens) | 6.446e-01 | 2.772e-22 | 2.740e-19 | 1.77e+02 | 1.78e+02 |
|  | [CGB7](https://www.ncbi.nlm.nih.gov/gene/?term=CGB7%5bSymbol%5d%20AND%20Homo%20sapiens) | 6.443e-01 | 2.953e-22 | 2.781e-19 | 1.77e+02 | 1.66e+02 |
|  | [TNFRSF12A](https://www.ncbi.nlm.nih.gov/gene/?term=TNFRSF12A%5bSymbol%5d%20AND%20Homo%20sapiens) | 6.402e-01 | 6.525e-22 | 5.865e-19 | 1.77e+02 | 1.78e+02 |
|  | [CST6](https://www.ncbi.nlm.nih.gov/gene/?term=CST6%5bSymbol%5d%20AND%20Homo%20sapiens) | 6.368e-01 | 1.246e-21 | 1.071e-18 | 1.77e+02 | 1.75e+02 |
|  | [S100A10](https://www.ncbi.nlm.nih.gov/gene/?term=S100A10%5bSymbol%5d%20AND%20Homo%20sapiens) | 6.361e-01 | 1.424e-21 | 1.154e-18 | 1.77e+02 | 1.78e+02 |
|  | [LAMB3](https://www.ncbi.nlm.nih.gov/gene/?term=LAMB3%5bSymbol%5d%20AND%20Homo%20sapiens) | 6.360e-01 | 1.459e-21 | 1.154e-18 | 1.77e+02 | 1.78e+02 |
| S100A4 | [S100A4](https://www.ncbi.nlm.nih.gov/gene/?term=S100A4%5bSymbol%5d%20AND%20Homo%20sapiens) | 1.000e+00 | 1.000e-20 | 1.000e-16 | 1.78e+02 | 1.78e+02 |
|  | [EMP3](https://www.ncbi.nlm.nih.gov/gene/?term=EMP3%5bSymbol%5d%20AND%20Homo%20sapiens) | 6.254e-01 | 1.035e-20 | 1.023e-16 | 1.78e+02 | 1.78e+02 |
|  | [S100A3](https://www.ncbi.nlm.nih.gov/gene/?term=S100A3%5bSymbol%5d%20AND%20Homo%20sapiens) | 5.754e-01 | 4.422e-17 | 2.863e-13 | 1.78e+02 | 1.78e+02 |
|  | [RNF149](https://www.ncbi.nlm.nih.gov/gene/?term=RNF149%5bSymbol%5d%20AND%20Homo%20sapiens) | 5.728e-01 | 6.562e-17 | 2.863e-13 | 1.78e+02 | 1.78e+02 |
|  | [S100A5](https://www.ncbi.nlm.nih.gov/gene/?term=S100A5%5bSymbol%5d%20AND%20Homo%20sapiens) | 5.722e-01 | 7.239e-17 | 2.863e-13 | 1.78e+02 | 1.75e+02 |
|  | [PDLIM7](https://www.ncbi.nlm.nih.gov/gene/?term=PDLIM7%5bSymbol%5d%20AND%20Homo%20sapiens) | 5.669e-01 | 1.588e-16 | 5.232e-13 | 1.78e+02 | 1.78e+02 |
|  | [TMSL3](https://www.ncbi.nlm.nih.gov/gene/?term=TMSL3%5bSymbol%5d%20AND%20Homo%20sapiens) | 5.641e-01 | 2.411e-16 | 6.812e-13 | 1.78e+02 | 1.78e+02 |
|  | [CATSPER1](https://www.ncbi.nlm.nih.gov/gene/?term=CATSPER1%5bSymbol%5d%20AND%20Homo%20sapiens) | 5.604e-01 | 4.140e-16 | 1.023e-12 | 1.78e+02 | 1.76e+02 |
|  | [LGALS1](https://www.ncbi.nlm.nih.gov/gene/?term=LGALS1%5bSymbol%5d%20AND%20Homo%20sapiens) | 5.581e-01 | 5.797e-16 | 1.274e-12 | 1.78e+02 | 1.78e+02 |
|  | [PLAUR](https://www.ncbi.nlm.nih.gov/gene/?term=PLAUR%5bSymbol%5d%20AND%20Homo%20sapiens) | 5.573e-01 | 6.535e-16 | 1.292e-12 | 1.78e+02 | 1.78e+02 |
|  | [ZBED2](https://www.ncbi.nlm.nih.gov/gene/?term=ZBED2%5bSymbol%5d%20AND%20Homo%20sapiens) | 5.526e-01 | 1.266e-15 | 2.276e-12 | 1.78e+02 | 1.73e+02 |
|  | [S100A11](https://www.ncbi.nlm.nih.gov/gene/?term=S100A11%5bSymbol%5d%20AND%20Homo%20sapiens) | 5.461e-01 | 3.169e-15 | 5.221e-12 | 1.78e+02 | 1.78e+02 |
|  | [ACTB](https://www.ncbi.nlm.nih.gov/gene/?term=ACTB%5bSymbol%5d%20AND%20Homo%20sapiens) | 5.451e-01 | 3.638e-15 | 5.534e-12 | 1.78e+02 | 1.78e+02 |
|  | [S100A2](https://www.ncbi.nlm.nih.gov/gene/?term=S100A2%5bSymbol%5d%20AND%20Homo%20sapiens) | 5.440e-01 | 4.212e-15 | 5.949e-12 | 1.78e+02 | 1.77e+02 |
|  | [GPR87](https://www.ncbi.nlm.nih.gov/gene/?term=GPR87%5bSymbol%5d%20AND%20Homo%20sapiens) | 5.417e-01 | 5.851e-15 | 7.713e-12 | 1.78e+02 | 1.70e+02 |
|  | [FSCN1](https://www.ncbi.nlm.nih.gov/gene/?term=FSCN1%5bSymbol%5d%20AND%20Homo%20sapiens) | 5.405e-01 | 6.853e-15 | 8.469e-12 | 1.78e+02 | 1.78e+02 |
|  | [PLAU](https://www.ncbi.nlm.nih.gov/gene/?term=PLAU%5bSymbol%5d%20AND%20Homo%20sapiens) | 5.398e-01 | 7.525e-15 | 8.753e-12 | 1.78e+02 | 1.78e+02 |
|  | [PADI1](https://www.ncbi.nlm.nih.gov/gene/?term=PADI1%5bSymbol%5d%20AND%20Homo%20sapiens) | 5.388e-01 | 8.605e-15 | 9.255e-12 | 1.78e+02 | 1.75e+02 |
|  | [TMSB10](https://www.ncbi.nlm.nih.gov/gene/?term=TMSB10%5bSymbol%5d%20AND%20Homo%20sapiens) | 5.386e-01 | 8.893e-15 | 9.255e-12 | 1.78e+02 | 1.78e+02 |
|  | [KRT16](https://www.ncbi.nlm.nih.gov/gene/?term=KRT16%5bSymbol%5d%20AND%20Homo%20sapiens) | 5.377e-01 | 9.938e-15 | 9.826e-12 | 1.78e+02 | 1.73e+02 |
|  | [CRABP2](https://www.ncbi.nlm.nih.gov/gene/?term=CRABP2%5bSymbol%5d%20AND%20Homo%20sapiens) | 5.371e-01 | 1.081e-14 | 1.017e-11 | 1.78e+02 | 1.78e+02 |
|  | [CNN2](https://www.ncbi.nlm.nih.gov/gene/?term=CNN2%5bSymbol%5d%20AND%20Homo%20sapiens) | 5.367e-01 | 1.147e-14 | 1.031e-11 | 1.78e+02 | 1.78e+02 |
|  | [MYL12A](https://www.ncbi.nlm.nih.gov/gene/?term=MYL12A%5bSymbol%5d%20AND%20Homo%20sapiens) | 5.354e-01 | 1.361e-14 | 1.170e-11 | 1.78e+02 | 1.78e+02 |
|  | [LAMB3](https://www.ncbi.nlm.nih.gov/gene/?term=LAMB3%5bSymbol%5d%20AND%20Homo%20sapiens) | 5.343e-01 | 1.578e-14 | 1.277e-11 | 1.78e+02 | 1.78e+02 |
|  | [PPAPDC1A](https://www.ncbi.nlm.nih.gov/gene/?term=PPAPDC1A%5bSymbol%5d%20AND%20Homo%20sapiens) | 5.341e-01 | 1.614e-14 | 1.277e-11 | 1.78e+02 | 1.77e+02 |
| S100A6 | [S100A6](https://www.ncbi.nlm.nih.gov/gene/?term=S100A6%5bSymbol%5d%20AND%20Homo%20sapiens) | 1.000e+00 | 1.000e-51 | 1.000e-47 | 1.78e+02 | 1.78e+02 |
|  | [S100A11](https://www.ncbi.nlm.nih.gov/gene/?term=S100A11%5bSymbol%5d%20AND%20Homo%20sapiens) | 8.514e-01 | 3.340e-51 | 3.302e-47 | 1.78e+02 | 1.78e+02 |
|  | [S100A16](https://www.ncbi.nlm.nih.gov/gene/?term=S100A16%5bSymbol%5d%20AND%20Homo%20sapiens) | 8.474e-01 | 2.865e-50 | 1.889e-46 | 1.78e+02 | 1.78e+02 |
|  | [S100A10](https://www.ncbi.nlm.nih.gov/gene/?term=S100A10%5bSymbol%5d%20AND%20Homo%20sapiens) | 8.366e-01 | 7.058e-48 | 3.489e-44 | 1.78e+02 | 1.78e+02 |
|  | [PHLDA2](https://www.ncbi.nlm.nih.gov/gene/?term=PHLDA2%5bSymbol%5d%20AND%20Homo%20sapiens) | 8.201e-01 | 1.553e-44 | 6.143e-41 | 1.78e+02 | 1.78e+02 |
|  | [TAGLN2](https://www.ncbi.nlm.nih.gov/gene/?term=TAGLN2%5bSymbol%5d%20AND%20Homo%20sapiens) | 8.142e-01 | 2.034e-43 | 6.705e-40 | 1.78e+02 | 1.78e+02 |
|  | [EFNA4](https://www.ncbi.nlm.nih.gov/gene/?term=EFNA4%5bSymbol%5d%20AND%20Homo%20sapiens) | 7.972e-01 | 1.984e-40 | 5.604e-37 | 1.78e+02 | 1.78e+02 |
|  | [MVP](https://www.ncbi.nlm.nih.gov/gene/?term=MVP%5bSymbol%5d%20AND%20Homo%20sapiens) | 7.967e-01 | 2.427e-40 | 5.999e-37 | 1.78e+02 | 1.78e+02 |
|  | [TSPO](https://www.ncbi.nlm.nih.gov/gene/?term=TSPO%5bSymbol%5d%20AND%20Homo%20sapiens) | 7.878e-01 | 6.767e-39 | 1.487e-35 | 1.78e+02 | 1.78e+02 |
|  | [HN1](https://www.ncbi.nlm.nih.gov/gene/?term=HN1%5bSymbol%5d%20AND%20Homo%20sapiens) | 7.847e-01 | 2.111e-38 | 4.175e-35 | 1.78e+02 | 1.78e+02 |
|  | [SIK3](https://www.ncbi.nlm.nih.gov/gene/?term=SIK3%5bSymbol%5d%20AND%20Homo%20sapiens) | -7.774e-01 | 2.771e-37 | 4.952e-34 | 1.78e+02 | 1.78e+02 |
|  | [C19orf33](https://www.ncbi.nlm.nih.gov/gene/?term=C19orf33%5bSymbol%5d%20AND%20Homo%20sapiens) | 7.770e-01 | 3.219e-37 | 4.952e-34 | 1.78e+02 | 1.77e+02 |
|  | [CLIC1](https://www.ncbi.nlm.nih.gov/gene/?term=CLIC1%5bSymbol%5d%20AND%20Homo%20sapiens) | 7.768e-01 | 3.503e-37 | 4.952e-34 | 1.78e+02 | 1.78e+02 |
|  | [TSPAN15](https://www.ncbi.nlm.nih.gov/gene/?term=TSPAN15%5bSymbol%5d%20AND%20Homo%20sapiens) | 7.768e-01 | 3.506e-37 | 4.952e-34 | 1.78e+02 | 1.78e+02 |
|  | [PLEK2](https://www.ncbi.nlm.nih.gov/gene/?term=PLEK2%5bSymbol%5d%20AND%20Homo%20sapiens) | 7.762e-01 | 4.195e-37 | 5.531e-34 | 1.78e+02 | 1.78e+02 |
|  | [TMSB10](https://www.ncbi.nlm.nih.gov/gene/?term=TMSB10%5bSymbol%5d%20AND%20Homo%20sapiens) | 7.736e-01 | 1.028e-36 | 1.270e-33 | 1.78e+02 | 1.78e+02 |
|  | [EPS8L1](https://www.ncbi.nlm.nih.gov/gene/?term=EPS8L1%5bSymbol%5d%20AND%20Homo%20sapiens) | 7.720e-01 | 1.777e-36 | 2.067e-33 | 1.78e+02 | 1.78e+02 |
|  | [RHOC](https://www.ncbi.nlm.nih.gov/gene/?term=RHOC%5bSymbol%5d%20AND%20Homo%20sapiens) | 7.683e-01 | 6.168e-36 | 6.776e-33 | 1.78e+02 | 1.78e+02 |
|  | [ANXA2](https://www.ncbi.nlm.nih.gov/gene/?term=ANXA2%5bSymbol%5d%20AND%20Homo%20sapiens) | 7.679e-01 | 7.119e-36 | 7.409e-33 | 1.78e+02 | 1.78e+02 |
|  | [KRT19](https://www.ncbi.nlm.nih.gov/gene/?term=KRT19%5bSymbol%5d%20AND%20Homo%20sapiens) | 7.670e-01 | 9.382e-36 | 9.275e-33 | 1.78e+02 | 1.78e+02 |
|  | [SDCBP2](https://www.ncbi.nlm.nih.gov/gene/?term=SDCBP2%5bSymbol%5d%20AND%20Homo%20sapiens) | 7.656e-01 | 1.493e-35 | 1.405e-32 | 1.78e+02 | 1.78e+02 |
|  | [POLD4](https://www.ncbi.nlm.nih.gov/gene/?term=POLD4%5bSymbol%5d%20AND%20Homo%20sapiens) | 7.646e-01 | 2.096e-35 | 1.884e-32 | 1.78e+02 | 1.78e+02 |
|  | [TMEM92](https://www.ncbi.nlm.nih.gov/gene/?term=TMEM92%5bSymbol%5d%20AND%20Homo%20sapiens) | 7.624e-01 | 4.165e-35 | 3.566e-32 | 1.78e+02 | 1.77e+02 |
|  | [KIAA1632](https://www.ncbi.nlm.nih.gov/gene/?term=KIAA1632%5bSymbol%5d%20AND%20Homo%20sapiens) | -7.623e-01 | 4.328e-35 | 3.566e-32 | 1.78e+02 | 1.78e+02 |
|  | [ADAMTSL5](https://www.ncbi.nlm.nih.gov/gene/?term=ADAMTSL5%5bSymbol%5d%20AND%20Homo%20sapiens) | 7.588e-01 | 1.346e-34 | 1.064e-31 | 1.78e+02 | 1.77e+02 |
| S100A10 | [S100A10](https://www.ncbi.nlm.nih.gov/gene/?term=S100A10%5bSymbol%5d%20AND%20Homo%20sapiens) | 1.000e+00 | 1.000e-64 | 1.000e-60 | 1.78e+02 | 1.78e+02 |
|  | [S100A11](https://www.ncbi.nlm.nih.gov/gene/?term=S100A11%5bSymbol%5d%20AND%20Homo%20sapiens) | 8.975e-01 | 1.831e-64 | 1.811e-60 | 1.78e+02 | 1.78e+02 |
|  | [S100A16](https://www.ncbi.nlm.nih.gov/gene/?term=S100A16%5bSymbol%5d%20AND%20Homo%20sapiens) | 8.793e-01 | 1.357e-58 | 8.948e-55 | 1.78e+02 | 1.78e+02 |
|  | [S100A6](https://www.ncbi.nlm.nih.gov/gene/?term=S100A6%5bSymbol%5d%20AND%20Homo%20sapiens) | 8.366e-01 | 7.058e-48 | 3.489e-44 | 1.78e+02 | 1.78e+02 |
|  | [ANXA2](https://www.ncbi.nlm.nih.gov/gene/?term=ANXA2%5bSymbol%5d%20AND%20Homo%20sapiens) | 8.275e-01 | 5.527e-46 | 2.186e-42 | 1.78e+02 | 1.78e+02 |
|  | [LAMB3](https://www.ncbi.nlm.nih.gov/gene/?term=LAMB3%5bSymbol%5d%20AND%20Homo%20sapiens) | 8.216e-01 | 7.919e-45 | 2.610e-41 | 1.78e+02 | 1.78e+02 |
|  | [PLEK2](https://www.ncbi.nlm.nih.gov/gene/?term=PLEK2%5bSymbol%5d%20AND%20Homo%20sapiens) | 8.184e-01 | 3.349e-44 | 9.461e-41 | 1.78e+02 | 1.78e+02 |
|  | [GJB3](https://www.ncbi.nlm.nih.gov/gene/?term=GJB3%5bSymbol%5d%20AND%20Homo%20sapiens) | 8.073e-01 | 3.673e-42 | 9.079e-39 | 1.78e+02 | 1.75e+02 |
|  | [SFN](https://www.ncbi.nlm.nih.gov/gene/?term=SFN%5bSymbol%5d%20AND%20Homo%20sapiens) | 7.974e-01 | 1.858e-40 | 4.083e-37 | 1.78e+02 | 1.76e+02 |
|  | [PVRL4](https://www.ncbi.nlm.nih.gov/gene/?term=PVRL4%5bSymbol%5d%20AND%20Homo%20sapiens) | 7.959e-01 | 3.354e-40 | 6.631e-37 | 1.78e+02 | 1.76e+02 |
|  | [KRT19](https://www.ncbi.nlm.nih.gov/gene/?term=KRT19%5bSymbol%5d%20AND%20Homo%20sapiens) | 7.855e-01 | 1.595e-38 | 2.868e-35 | 1.78e+02 | 1.78e+02 |
|  | [EFNA4](https://www.ncbi.nlm.nih.gov/gene/?term=EFNA4%5bSymbol%5d%20AND%20Homo%20sapiens) | 7.844e-01 | 2.331e-38 | 3.841e-35 | 1.78e+02 | 1.78e+02 |
|  | [AIM1L](https://www.ncbi.nlm.nih.gov/gene/?term=AIM1L%5bSymbol%5d%20AND%20Homo%20sapiens) | 7.829e-01 | 3.994e-38 | 5.904e-35 | 1.78e+02 | 1.77e+02 |
|  | [C19orf33](https://www.ncbi.nlm.nih.gov/gene/?term=C19orf33%5bSymbol%5d%20AND%20Homo%20sapiens) | 7.828e-01 | 4.180e-38 | 5.904e-35 | 1.78e+02 | 1.77e+02 |
|  | [EPS8L1](https://www.ncbi.nlm.nih.gov/gene/?term=EPS8L1%5bSymbol%5d%20AND%20Homo%20sapiens) | 7.825e-01 | 4.637e-38 | 6.113e-35 | 1.78e+02 | 1.78e+02 |
|  | [LGALS3](https://www.ncbi.nlm.nih.gov/gene/?term=LGALS3%5bSymbol%5d%20AND%20Homo%20sapiens) | 7.823e-01 | 5.084e-38 | 6.283e-35 | 1.78e+02 | 1.78e+02 |
|  | [CAPG](https://www.ncbi.nlm.nih.gov/gene/?term=CAPG%5bSymbol%5d%20AND%20Homo%20sapiens) | 7.817e-01 | 6.260e-38 | 6.929e-35 | 1.78e+02 | 1.78e+02 |
|  | [TAGLN2](https://www.ncbi.nlm.nih.gov/gene/?term=TAGLN2%5bSymbol%5d%20AND%20Homo%20sapiens) | 7.817e-01 | 6.308e-38 | 6.929e-35 | 1.78e+02 | 1.78e+02 |
|  | [KCNN4](https://www.ncbi.nlm.nih.gov/gene/?term=KCNN4%5bSymbol%5d%20AND%20Homo%20sapiens) | 7.799e-01 | 1.163e-37 | 1.210e-34 | 1.78e+02 | 1.78e+02 |
|  | [GPRC5A](https://www.ncbi.nlm.nih.gov/gene/?term=GPRC5A%5bSymbol%5d%20AND%20Homo%20sapiens) | 7.786e-01 | 1.874e-37 | 1.853e-34 | 1.78e+02 | 1.78e+02 |
|  | [ANXA2P2](https://www.ncbi.nlm.nih.gov/gene/?term=ANXA2P2%5bSymbol%5d%20AND%20Homo%20sapiens) | 7.760e-01 | 4.546e-37 | 4.281e-34 | 1.78e+02 | 1.78e+02 |
|  | [MST1R](https://www.ncbi.nlm.nih.gov/gene/?term=MST1R%5bSymbol%5d%20AND%20Homo%20sapiens) | 7.708e-01 | 2.674e-36 | 2.318e-33 | 1.78e+02 | 1.78e+02 |
|  | [FLJ23867](https://www.ncbi.nlm.nih.gov/gene/?term=FLJ23867%5bSymbol%5d%20AND%20Homo%20sapiens) | 7.708e-01 | 2.696e-36 | 2.318e-33 | 1.78e+02 | 1.78e+02 |
|  | [TMEM92](https://www.ncbi.nlm.nih.gov/gene/?term=TMEM92%5bSymbol%5d%20AND%20Homo%20sapiens) | 7.697e-01 | 3.880e-36 | 3.197e-33 | 1.78e+02 | 1.77e+02 |
|  | [IGSF9](https://www.ncbi.nlm.nih.gov/gene/?term=IGSF9%5bSymbol%5d%20AND%20Homo%20sapiens) | 7.680e-01 | 6.890e-36 | 5.450e-33 | 1.78e+02 | 1.77e+02 |
| S100A14 | [S100A14](https://www.ncbi.nlm.nih.gov/gene/?term=S100A14%5bSymbol%5d%20AND%20Homo%20sapiens) | 1.000e+00 | 1.000e-58 | 1.000e-55 | 1.76e+02 | 1.76e+02 |
|  | [PVRL4](https://www.ncbi.nlm.nih.gov/gene/?term=PVRL4%5bSymbol%5d%20AND%20Homo%20sapiens) | 8.797e-01 | 1.011e-58 | 9.996e-55 | 1.76e+02 | 1.76e+02 |
|  | [C1orf106](https://www.ncbi.nlm.nih.gov/gene/?term=C1orf106%5bSymbol%5d%20AND%20Homo%20sapiens) | 8.760e-01 | 1.223e-57 | 8.060e-54 | 1.76e+02 | 1.77e+02 |
|  | [SFN](https://www.ncbi.nlm.nih.gov/gene/?term=SFN%5bSymbol%5d%20AND%20Homo%20sapiens) | 8.663e-01 | 6.222e-55 | 3.076e-51 | 1.76e+02 | 1.76e+02 |
|  | [PRSS8](https://www.ncbi.nlm.nih.gov/gene/?term=PRSS8%5bSymbol%5d%20AND%20Homo%20sapiens) | 8.632e-01 | 3.902e-54 | 1.543e-50 | 1.76e+02 | 1.78e+02 |
|  | [S100A16](https://www.ncbi.nlm.nih.gov/gene/?term=S100A16%5bSymbol%5d%20AND%20Homo%20sapiens) | 8.626e-01 | 5.651e-54 | 1.862e-50 | 1.76e+02 | 1.78e+02 |
|  | [LAMB3](https://www.ncbi.nlm.nih.gov/gene/?term=LAMB3%5bSymbol%5d%20AND%20Homo%20sapiens) | 8.534e-01 | 1.140e-51 | 3.221e-48 | 1.76e+02 | 1.78e+02 |
|  | [TMPRSS4](https://www.ncbi.nlm.nih.gov/gene/?term=TMPRSS4%5bSymbol%5d%20AND%20Homo%20sapiens) | 8.508e-01 | 4.526e-51 | 1.119e-47 | 1.76e+02 | 1.75e+02 |
|  | [PLEK2](https://www.ncbi.nlm.nih.gov/gene/?term=PLEK2%5bSymbol%5d%20AND%20Homo%20sapiens) | 8.462e-01 | 5.496e-50 | 1.207e-46 | 1.76e+02 | 1.78e+02 |
|  | [TMEM92](https://www.ncbi.nlm.nih.gov/gene/?term=TMEM92%5bSymbol%5d%20AND%20Homo%20sapiens) | 8.459e-01 | 6.254e-50 | 1.237e-46 | 1.76e+02 | 1.77e+02 |
|  | [MST1R](https://www.ncbi.nlm.nih.gov/gene/?term=MST1R%5bSymbol%5d%20AND%20Homo%20sapiens) | 8.439e-01 | 1.796e-49 | 3.228e-46 | 1.76e+02 | 1.78e+02 |
|  | [EFNA4](https://www.ncbi.nlm.nih.gov/gene/?term=EFNA4%5bSymbol%5d%20AND%20Homo%20sapiens) | 8.374e-01 | 4.770e-48 | 7.859e-45 | 1.76e+02 | 1.78e+02 |
|  | [LAD1](https://www.ncbi.nlm.nih.gov/gene/?term=LAD1%5bSymbol%5d%20AND%20Homo%20sapiens) | 8.295e-01 | 2.193e-46 | 3.336e-43 | 1.76e+02 | 1.78e+02 |
|  | [S100A11](https://www.ncbi.nlm.nih.gov/gene/?term=S100A11%5bSymbol%5d%20AND%20Homo%20sapiens) | 8.258e-01 | 1.216e-45 | 1.632e-42 | 1.76e+02 | 1.78e+02 |
|  | [ANXA3](https://www.ncbi.nlm.nih.gov/gene/?term=ANXA3%5bSymbol%5d%20AND%20Homo%20sapiens) | 8.257e-01 | 1.238e-45 | 1.632e-42 | 1.76e+02 | 1.78e+02 |
|  | [C6orf132](https://www.ncbi.nlm.nih.gov/gene/?term=C6orf132%5bSymbol%5d%20AND%20Homo%20sapiens) | 8.185e-01 | 3.213e-44 | 3.971e-41 | 1.76e+02 | 1.77e+02 |
|  | [PDZK1IP1](https://www.ncbi.nlm.nih.gov/gene/?term=PDZK1IP1%5bSymbol%5d%20AND%20Homo%20sapiens) | 8.162e-01 | 8.467e-44 | 9.848e-41 | 1.76e+02 | 1.78e+02 |
|  | [FUT3](https://www.ncbi.nlm.nih.gov/gene/?term=FUT3%5bSymbol%5d%20AND%20Homo%20sapiens) | 8.140e-01 | 2.187e-43 | 2.403e-40 | 1.76e+02 | 1.77e+02 |
|  | [GJB3](https://www.ncbi.nlm.nih.gov/gene/?term=GJB3%5bSymbol%5d%20AND%20Homo%20sapiens) | 8.132e-01 | 3.155e-43 | 3.283e-40 | 1.76e+02 | 1.75e+02 |
|  | [ITGB4](https://www.ncbi.nlm.nih.gov/gene/?term=ITGB4%5bSymbol%5d%20AND%20Homo%20sapiens) | 8.103e-01 | 1.062e-42 | 1.050e-39 | 1.76e+02 | 1.78e+02 |
|  | [C19orf33](https://www.ncbi.nlm.nih.gov/gene/?term=C19orf33%5bSymbol%5d%20AND%20Homo%20sapiens) | 8.101e-01 | 1.118e-42 | 1.053e-39 | 1.76e+02 | 1.77e+02 |
|  | [LGALS3](https://www.ncbi.nlm.nih.gov/gene/?term=LGALS3%5bSymbol%5d%20AND%20Homo%20sapiens) | 8.048e-01 | 1.005e-41 | 9.033e-39 | 1.76e+02 | 1.78e+02 |
|  | [EPS8L1](https://www.ncbi.nlm.nih.gov/gene/?term=EPS8L1%5bSymbol%5d%20AND%20Homo%20sapiens) | 8.028e-01 | 2.191e-41 | 1.884e-38 | 1.76e+02 | 1.78e+02 |
|  | [PPAP2C](https://www.ncbi.nlm.nih.gov/gene/?term=PPAP2C%5bSymbol%5d%20AND%20Homo%20sapiens) | 7.998e-01 | 7.303e-41 | 6.017e-38 | 1.76e+02 | 1.78e+02 |
|  | [KRT19](https://www.ncbi.nlm.nih.gov/gene/?term=KRT19%5bSymbol%5d%20AND%20Homo%20sapiens) | 7.988e-01 | 1.069e-40 | 8.457e-38 | 1.76e+02 | 1.78e+02 |
| S100A16 | [S100A16](https://www.ncbi.nlm.nih.gov/gene/?term=S100A16%5bSymbol%5d%20AND%20Homo%20sapiens) | 1.000e+00 | 1.000e-71 | 1.000e-67 | 1.78e+02 | 1.78e+02 |
|  | [S100A11](https://www.ncbi.nlm.nih.gov/gene/?term=S100A11%5bSymbol%5d%20AND%20Homo%20sapiens) | 9.151e-01 | 2.462e-71 | 2.434e-67 | 1.78e+02 | 1.78e+02 |
|  | [S100A10](https://www.ncbi.nlm.nih.gov/gene/?term=S100A10%5bSymbol%5d%20AND%20Homo%20sapiens) | 8.793e-01 | 1.357e-58 | 8.948e-55 | 1.78e+02 | 1.78e+02 |
|  | [LAMB3](https://www.ncbi.nlm.nih.gov/gene/?term=LAMB3%5bSymbol%5d%20AND%20Homo%20sapiens) | 8.731e-01 | 8.443e-57 | 4.174e-53 | 1.78e+02 | 1.78e+02 |
|  | [S100A14](https://www.ncbi.nlm.nih.gov/gene/?term=S100A14%5bSymbol%5d%20AND%20Homo%20sapiens) | 8.626e-01 | 5.651e-54 | 2.235e-50 | 1.78e+02 | 1.76e+02 |
|  | [SFN](https://www.ncbi.nlm.nih.gov/gene/?term=SFN%5bSymbol%5d%20AND%20Homo%20sapiens) | 8.544e-01 | 6.439e-52 | 2.122e-48 | 1.78e+02 | 1.76e+02 |
|  | [PLEK2](https://www.ncbi.nlm.nih.gov/gene/?term=PLEK2%5bSymbol%5d%20AND%20Homo%20sapiens) | 8.485e-01 | 1.613e-50 | 4.556e-47 | 1.78e+02 | 1.78e+02 |
|  | [EFNA4](https://www.ncbi.nlm.nih.gov/gene/?term=EFNA4%5bSymbol%5d%20AND%20Homo%20sapiens) | 8.482e-01 | 1.870e-50 | 4.622e-47 | 1.78e+02 | 1.78e+02 |
|  | [S100A6](https://www.ncbi.nlm.nih.gov/gene/?term=S100A6%5bSymbol%5d%20AND%20Homo%20sapiens) | 8.474e-01 | 2.865e-50 | 6.295e-47 | 1.78e+02 | 1.78e+02 |
|  | [ANXA2](https://www.ncbi.nlm.nih.gov/gene/?term=ANXA2%5bSymbol%5d%20AND%20Homo%20sapiens) | 8.440e-01 | 1.700e-49 | 3.362e-46 | 1.78e+02 | 1.78e+02 |
|  | [KRT19](https://www.ncbi.nlm.nih.gov/gene/?term=KRT19%5bSymbol%5d%20AND%20Homo%20sapiens) | 8.403e-01 | 1.135e-48 | 2.040e-45 | 1.78e+02 | 1.78e+02 |
|  | [ITGB4](https://www.ncbi.nlm.nih.gov/gene/?term=ITGB4%5bSymbol%5d%20AND%20Homo%20sapiens) | 8.373e-01 | 5.186e-48 | 8.545e-45 | 1.78e+02 | 1.78e+02 |
|  | [MDFI](https://www.ncbi.nlm.nih.gov/gene/?term=MDFI%5bSymbol%5d%20AND%20Homo%20sapiens) | 8.291e-01 | 2.608e-46 | 3.967e-43 | 1.78e+02 | 1.78e+02 |
|  | [GJB3](https://www.ncbi.nlm.nih.gov/gene/?term=GJB3%5bSymbol%5d%20AND%20Homo%20sapiens) | 8.276e-01 | 5.240e-46 | 7.401e-43 | 1.78e+02 | 1.75e+02 |
|  | [TUBA1C](https://www.ncbi.nlm.nih.gov/gene/?term=TUBA1C%5bSymbol%5d%20AND%20Homo%20sapiens) | 8.207e-01 | 1.175e-44 | 1.549e-41 | 1.78e+02 | 1.78e+02 |
|  | [TMEM92](https://www.ncbi.nlm.nih.gov/gene/?term=TMEM92%5bSymbol%5d%20AND%20Homo%20sapiens) | 8.205e-01 | 1.295e-44 | 1.600e-41 | 1.78e+02 | 1.77e+02 |
|  | [PVRL4](https://www.ncbi.nlm.nih.gov/gene/?term=PVRL4%5bSymbol%5d%20AND%20Homo%20sapiens) | 8.194e-01 | 2.156e-44 | 2.508e-41 | 1.78e+02 | 1.76e+02 |
|  | [KCNN4](https://www.ncbi.nlm.nih.gov/gene/?term=KCNN4%5bSymbol%5d%20AND%20Homo%20sapiens) | 8.163e-01 | 8.383e-44 | 9.209e-41 | 1.78e+02 | 1.78e+02 |
|  | [GSTP1](https://www.ncbi.nlm.nih.gov/gene/?term=GSTP1%5bSymbol%5d%20AND%20Homo%20sapiens) | 8.126e-01 | 4.024e-43 | 4.188e-40 | 1.78e+02 | 1.78e+02 |
|  | [EPS8L1](https://www.ncbi.nlm.nih.gov/gene/?term=EPS8L1%5bSymbol%5d%20AND%20Homo%20sapiens) | 8.105e-01 | 9.686e-43 | 9.577e-40 | 1.78e+02 | 1.78e+02 |
|  | [C1orf106](https://www.ncbi.nlm.nih.gov/gene/?term=C1orf106%5bSymbol%5d%20AND%20Homo%20sapiens) | 8.101e-01 | 1.148e-42 | 1.081e-39 | 1.78e+02 | 1.77e+02 |
|  | [LGALS3](https://www.ncbi.nlm.nih.gov/gene/?term=LGALS3%5bSymbol%5d%20AND%20Homo%20sapiens) | 8.085e-01 | 2.179e-42 | 1.958e-39 | 1.78e+02 | 1.78e+02 |
|  | [RHBDF1](https://www.ncbi.nlm.nih.gov/gene/?term=RHBDF1%5bSymbol%5d%20AND%20Homo%20sapiens) | 8.081e-01 | 2.651e-42 | 2.279e-39 | 1.78e+02 | 1.78e+02 |
|  | [MST1R](https://www.ncbi.nlm.nih.gov/gene/?term=MST1R%5bSymbol%5d%20AND%20Homo%20sapiens) | 8.076e-01 | 3.176e-42 | 2.617e-39 | 1.78e+02 | 1.78e+02 |
|  | [TMSB10](https://www.ncbi.nlm.nih.gov/gene/?term=TMSB10%5bSymbol%5d%20AND%20Homo%20sapiens) | 8.066e-01 | 4.837e-42 | 3.825e-39 | 1.78e+02 | 1.78e+02 |

1. P-value , P-value obtained from statistical method.

2. FDR (BH), FDR is calculated by BH (Benjamini-Hochberg method).

3. Event_SD, Number of observations in search dataset attribute without NA’s and Zero’s.

4. Event_TD, Number of observations in target dataset attribute without NA’s and Zero’s.
