# Supplementary material for: Comprehensive analysis of the transcriptional expressions and prognostic value of S100A family in pancreatic ductal adenocarcinoma
Source: BMC Cancer. 2021 Sep 16;21:1039. doi: 10.1186/s12885-021-08769-6 (PMC8447682; doi:10.1186/s12885-021-08769-6)
Supplement: Supplementary file 3 — Additional file 3: Supplementary Table S3–8. Basic characteristics of 43 PDAC patients. [file 12885_2021_8769_MOESM3_ESM.zip › Supplementary Table S4.docx]

| Characteristic | S100A4 low  (n = 20) | S100A4 high  (n = 23) | P value |
| --- | --- | --- | --- |
| Age |  |  | 0.780 |
| ≥ 55 | 13 | 14 |  |
| < 55 | 7 | 9 |  |
| Sex |  |  | 0.526 |
| Male | 13 | 17 |  |
| Female | 7 | 6 |  |
| Differentiated degree |  |  | 0.391 |
| Well differentiated | 2 | 2 |  |
| Moderately differentiated | 14 | 12 |  |
| Poorly differentiated | 4 | 9 |  |
| Tumor size |  |  | 0.526 |
| ≥ 5 | 13 | 17 |  |
| < 5 | 7 | 6 |  |
| Depth of invasion |  |  | 0.947 |
| T1-2 | 12 | 13 |  |
| T3 | 6 | 7 |  |
| T4 | 2 | 3 |  |
| Lymph node |  |  | 0.853 |
| No-1 | 17 | 20 |  |
| N2-3 | 3 | 3 |  |

Table S4 Basic characteristics of 43 PDAC patients
